# Enzymatic Production of Prebiotic Xylooligosaccharides Using a *Bacillus pumilus* GH30_8 Glucuronoxylanase: Structural Basis of Glucuronoxylan Recognition and Hydrolysis

**DOI:** 10.1021/acs.jafc.5c07569

**Published:** 2026-02-03

**Authors:** Milena Moreira Vacilotto, Vanessa de Oliveira Arnoldi Pellegrini, Evandro Aresde de Araujo, Marcelo V. Liberato, Igor Polikarpov

**Affiliations:** † Instituto de Física de São Carlos, 42512 Universidade de São Paulo, Avenida Trabalhador São-Carlense 400, São Carlos, SP 13566-590, Brazil; ‡ Brazilian Synchrotron Light Laboratory (LNLS), Brazilian Center for Research in Energy and Materials (CNPEM), Giuseppe Máximo Scolfaro 10000, Campinas, SP 13083-100, Brazil

**Keywords:** xylanase, GH30, XOS, prebiotics

## Abstract

Transformation of agro-industrial products into value-added products, such as prebiotic oligosaccharides, is a key element of the emerging bioeconomy. Here, we characterized a new GH30_8 glucuronoxylanase from *Bacillus pumilus* (*Bp*Xyn30_8A) for its potential in producing xylooligosaccharides (XOS). *Bp*Xyn30_8A showed tolerance to ethanol and NaCl and released both linear and branched XOS containing MeGlcA at the penultimate nonreducing end residue. Its X-ray structure, determined at 2.16 Å resolution, revealed high similarity to other glucuronoxylanases. Furthermore, *Bp*Xyn30_8A achieved higher xylan conversion yields from corn cob and *Eucalyptus* sawdust than *Ruminococcus champanellensis* *Rc*Xyn30A. Finally, fermentation assays showed that *Bifidobacterium adolescentis* metabolized neutral XOS to acetate and lactate, whereas acidic XOS were poorly utilized. These results highlight the potential of *Bp*Xyn30_8A as a valuable enzyme for the green transformation of plant biomass into prebiotic oligosaccharides with promising applications in human and animal nutrition, health, and biotechnology.

## Graphical abstract

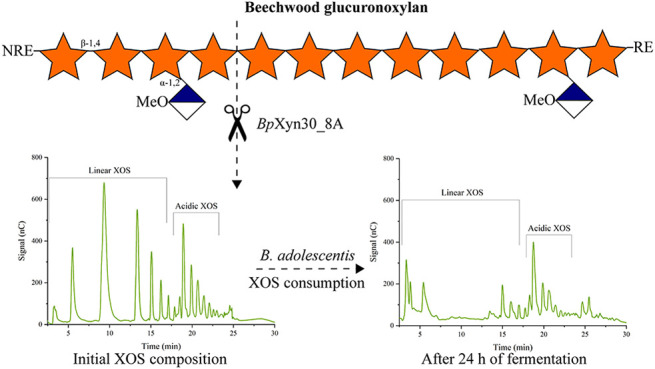

## 1 Introduction

In the last few decades, considerable attention has been given to the valorization of plant biomass residues, not only as a means to reduce the consumption of nonrenewable fossil fuels but also to promote the management of agro-industrial wastes.^1^ This approach aims for the optimization of the value obtained from crops, such as the carbohydrate-rich commodities (corn, wheat, and sugarcane) for the production of starch and sugar, and the sustainable conversion of their residue streams into second-generation products. Among the latter products with commercial interest, second-generation biofuels, renewable chemical compounds and materials, and prebiotic oligosaccharides stand out.^2,3^

The lignocellulosic biomass is highly recalcitrant. Its chemical composition mainly consists of biopolymers, such as cellulose and hemicelluloses. Lignin, proteins, metabolites, and inorganic compounds also make up part of the plant biomass. The proportion of components may vary significantly from plant to plant. All parts strongly interlace, resulting in an intricate and resistant structure.^4^ In agricultural crops (corn, sugarcane, and wheat) and in hardwoods (*Eucalyptus* and beechwood), xylan is the main form of hemicellulose. β-1,4-linked xylopyranose residues constitute a backbone chain of xylan, which is frequently decorated.^5^

The valorization of these feedstocks into higher-value products often involves carbohydrate-active enzymes (CAZymes), which transform complex carbohydrate biopolymers into simple sugars.^6^ Within glycoside hydrolase family 30 (GH30), only subfamilies GH30_7 (EC 3.2.1.8) and GH30_8 (EC 3.2.1.136) contain xylanases. GH30_7 primarily comprises eukaryotic enzymes with broader substrate specificity, while GH30_8 consists mainly of prokaryotic glucuronoxylanases. These GH30_8 enzymes specifically target glucuronoxylan, a type of xylan decorated with glucuronic acid (GlcA) or i4-O-methylated glucuronic acid (MeGlcA) at the C2 position of xylopyranosyl (Xylp) residues. However, more promiscuous enzymes with activity against arabinose-substituted xylans (glucuronoarabinoxylan endo-β-1,4-xylosidase) have also been reported within this subfamily.^7^

The enzymatic hydrolysis of biomass is a more sustainable and greener method for the disruption of plant cell fibers, as it can be carried out under milder conditions and does not produce unwanted byproducts. But, thermochemical pretreatments are frequently necessary in order to reduce biomass recalcitrance and improve the accessibility of the substrates to the enzymes.^8^ The alkaline pretreatment, for instance, uses chemicals, such as sodium hydroxide (NaOH), to solubilize the lignin.^9^

Products, such as xylitol, sorbitol, alcohol, furfural, and oligosaccharides, can be obtained from the hemicellulosic part of the biomass.^5^ Oligosaccharides look particularly attractive because of their prospective uses in the pharmaceutical and cosmetic industries, as well as for human and animal nutrition. A number of plant-derived oligosaccharides have prebiotic properties, capable to stimulate the growth of beneficial bacteria in the gastrointestinal tract, including *Bifidobacterium* sp. and *Lactobacillus* sp.^10,11^

Xylooligosaccharides (XOS), nondigestible sugar oligomers composed of 2 to 10 xylose units, are highly resistant to the harsh environments of the gastrointestinal tract and have been shown to promote host health when incorporated into the diet.^12,13^ The mechanism of the prebiotic response is still a subject of study, but it is known that their consumption by probiotic bacteria leads to a pH decrease as a result of the production of short-chain fatty acids (SCFAs), which prevents the growth of pathogenic bacteria and stimulates a variety of health responses, such as immune response, cholesterol reduction, anticancer activity, etc.^13^ At present, it is not completely clear if the growth of probiotic bacteria is influenced by oligosaccharide decorations.

Herein, we describe the enzymatic characterization of a novel *Bacillus pumilus* GH30_8 glucuronoxylanase (*Bp*Xyn30_8A), a mesophilic bacterium found in the most diverse terrestrial and marine settings,^14^ and applied the enzyme for the production of xylooligosaccharides from commercial substrates and lignocellulosic biomass. We also determined the *Bp*Xyn30_8A crystallographic structure in order to provide structural insights into substrate binding and the enzyme’s cleavage pattern. Lastly, to demonstrate the prebiotic activity, XOS produced from glucuronoxylan were used as a carbon source for *Bifidobacterium adolescentis* and the fermentation products were analyzed.

## 2 Material and Methods

### 2.1 Enzyme Selection, Cloning, Heterologous Expression, and Purification

The amino acid sequences of CAZymes from the microorganism gDNAs available in our group (Sao Carlos Institute of Physics, University of São Paulo, Brazil) were submitted to the Conserved Domain Search (CD-Search)^15^ and BLAST (http://blast.ncbi.nlm.nih.gov/Blast.cgi?PAGE=Proteins) to identify potential CAZyme targets.

The open reading frame (ORF) of *Bacillus pumilus* glucuronoxylanase from glycoside hydrolase family GH30_8 (*Bp*Xyn30_8A) (GenBank ID: WP_034619861.1), devoid of its signal peptide, was amplified by Polymerase Chain Reaction (PCR) using the gDNA of the bacteria, then cloned into the pETTRXA-1a expression vector using the Ligation-Independent Cloning (LIC) method, as established previously.^16^ The primers were as follows (LIC regions are highlighted in bold): forward 5′–**CAGGGCGCCATG**GCAAGTGATGCGAATATTAATG–3′ and reverse 5′–**GACCCGACGCGGTTA**GCGTTTGACCACAAAT–3′. This construction contained a 6xHis-thioredoxin tag from *E. coli* ^17^ at the N-terminal of the protein sequence, preceded by a Tobacco Etch Virus (TEV) cleavage site, and a kanamycin resistance marker. After plasmid propagation in *Escherichia coli* DH5α cells (Invitrogen, Massachusetts, USA), the purified vector containing the insert was transformed into competent *E. coli* Rosetta cells (DE3) (Invitrogen, Massachusetts, USA), which contain a chloramphenicol-resistant plasmid. All cloning steps were conducted following previously determined protocols.^18^

Positive *E. coli* Rosetta (DE3) transformants were grown at 37 °C in LB medium, which contained kanamycin (50 μg/mL) and chloramphenicol (34 μg/mL), until the OD_600_ reached 0.6, followed by inoculum induction with 0.5 mM isopropyl β-d-1-thiogalactopyranoside (IPTG) at 30 °C for 20 h. Centrifugation at 7,878 *g* for 30 min at 4 °C was applied to harvest the cells, which were then resuspended in 50 mM Tris-HCl buffer (pH 7.5) containing 500 mM NaCl, 1 mM dithiothreitol (DTT), and 1 mM phenylmethylsulfonyl fluoride (PMSF), and lysed by ultrasound waves (six cycles of 30 s on and off at 40% amplitude) using an F550 Sonic Dismembrator (Fisher Scientific, Hampton, USA). The supernatant was recovered after the sample was submitted to centrifugation at 13000 × *g* for 30 min at 4 °C, and the first protein purification was carried out using the Ni-NTA Superflow resin (Qiagen, Hilden, Germany) in 50 mM Tris-HCl buffer at pH 7.5 and 150 mM NaCl. The enzyme was eluted using buffers containing 50 mM Tris-HCl (pH 7.5), 150 mM NaCl, and increasing concentrations of imidazole (from 10 to 500 mM).

To remove the imidazole, the protein fractions containing *Bp*Xyn30_8A were subjected to dialysis, and the 6xHis-thioredoxin tag of the protein was cleaved with 40 μg/mL TEV and 1 mM DTT overnight at 4 °C. Finally, the enzyme was submitted to another affinity chromatography using Ni-NTA resin, and tag-free glucuronoxylanase was eluted using 50 mM Tris-HCl buffer at pH 7.5 and 150 mM NaCl. The efficiency of the enzyme purification was evaluated using 15% SDS-PAGE gels, and the concentration of the purified enzyme was measured by a NanoDrop 2000 Spectrophotometer (Thermo Scientific, Waltham, USA) at 280 nm wavelength. The enzyme’s theoretical mass (44.4 kDa) and molar extinction coefficient (92.8 M^–1^·cm^–1^) were used.

In addition, *Ruminococcus champanellensis* glucuronoxylanase from family GH30_8 (*Rc*Xyn30_8A) was obtained as previously described.^18^

### 2.2 Differential Scanning Fluorimetry

In order to evaluate the optimal conditions for the preservation of glucuronoxylanase, *Bp*Xyn30_8A structural stability was evaluated by differential scanning fluorimetry (DSF)^19,20^ in the presence of 48 different buffers with pHs ranging from 1.2 to 10. The experimental mixture consisted of 20 μL of the enzyme at 0.37 mg/mL in 50 mM buffer, with or without 150 mM NaCl and 1x diluted SYPRO Orange dye (Invitrogen, Carlsbad, USA). The Microseal “B” seal (Bio-Rad, Hercules, USA) was used to seal a 96-well PCR plate, which was then incubated in a CFX96 Real-Time PCR Detection System (Bio-Rad, Hercules, USA). The scanning temperature was changed from 25 to 95 °C, with a 1 °C step every 30 s, and the SYPRO Orange dye extrinsic fluorescence was evaluated using 490/530 nm excitation/emission wavelengths. The curves’ derivative, calculated by the Bio-Rad CFX Manager software, was applied to determine the enzyme melting temperature (*T*_m_) for each tested condition.

### 2.3 Enzymatic Assays

Enzymatic assays were performed by the detection of reducing sugars using the 3,5-dinitrosalicylic acid (DNS) method and d-(+)-xylose for calibration. Reactions were prepared in triplicate and conducted at 55 °C for 12 min with 180 nM *Bp*Xyn30_8A in 20 mM Tris-HCl buffer at pH 7 and 0.5% (w/v) beechwood 4-O-methyl-glucuronoxylan, except when stated otherwise. After the incubation time, the samples were diluted once with DNS and heated at 95 °C for 5 min.

The enzyme specific activity (units per milligram) was evaluated in the presence of a variety of putative substrates: Avicel and carboxymethylcellulose (both from Sigma-Aldrich, St. Louis, USA); β-glucan, arabinan, xyloglucan, lichenan, rye arabinoxylan, and beechwood glucuronoxylan (all from Megazyme, Wicklow, Republic of Ireland). The definition of enzymatic activity (*U*) is the amount of products produced per minute per milligram of enzyme, expressed in units of μmol·min^–1^·mg^–1^. As the glucuronoxylanase exhibited activity exclusively toward beechwood glucuronoxylan, this substrate was selected for the characterization assays.

Optimal pH and temperature of the glucuronoxylanase were obtained by fixing one of the parameters and varying the other: for the former, 40 mM acetate/borate phosphate (ABF) buffer with pHs between 2 and 10 at 50 °C was employed, whereas for the latter, the temperature was varied from 20 to 80 °C at the enzyme’s optimal pH of 7 in 50 mM Tris-HCl buffer.

The enzyme thermal stability was evaluated by preincubating the protein at 50 or 55 °C and removing aliquots over time to test its activity using the DNS assay. The glucuronoxylanase half-life (*t*_1/2_) was calculated by linearizing the curve of relative activity (%) vs time by using the natural logarithm scale on the *y*-axis, and its slope (decay constant λ) was substituted into eq 1. Furthermore, *Bp*Xyn30_8A tolerance to salt and ethanol was evaluated by maintaining the enzyme for 1 h at room temperature in solutions containing 0 to 20% (v/v) ethanol or 0 to 2.8 M NaCl in Tris-HCl buffer (20 mM, pH 7). Residual activity was determined using DNS.

1 $$t_{1/2}=\frac{\ln\left(2\right)}{\lambda }$$

Since both *Rc*Xyn30_8A^18^ and *Bp*Xyn30_8A displayed activity against glucuronoxylan only, their kinetic parameters were determined using this substrate by increasing its concentration up to 12 g/L and incubating it with either *Rc*Xyn30A (at 15.6 nM) in 20 mM sodium phosphate buffer for 7 min at 50 °C or *Bp*Xyn30_8A (at 180 nM) in Tris-HCl buffer (20 mM, pH 7) for 12 min at 55 °C. The data were analyzed with the Michaelis-Menten fitting using OriginLab software (Version 2020).

### 2.4 Enzymatic Cleavage Pattern Analysis

Soluble products released by *Bp*Xyn30_8A after incubation with 0.5% (w/v) beechwood xylan and/or xylotetraose (X4), xylopentaose (X5), or xylohexaose (X6) (Megazyme, Wicklow, Ireland) at concentrations of 0.05 and/or 0.5 mg/mL were evaluated by high-performance anion exchange chromatography with pulsed amperometric detection (HPAEC-PAD) using the Dionex ICS-5000 system equipped with a CarboPAC1 guard column (2 mm × 50 mm) and a CarboPAC1 analytical column (2 mm × 250 mm) (Thermo Scientific, Waltham, USA). The reactions were prepared in 2 mL tubes under the same conditions described in Section 2.3, and incubated in a ThermoMixer C (Eppendorf, Hamburg, Germany) at 1000 rpm and 55 °C. The samples were taken over the course of 24 h, maintained at 95 °C for 10 min to denature the enzyme, and filtered with CHROMAFIL Xtra PTFE-20/25 syringe filters (Macherey-Nagel, Düren, Germany) before the HPAEC-PAD analysis. 100 mM NaOH (buffer A) and 1 M sodium acetate with 100 mM NaOH (buffer B) were utilized as eluents. The HPAEC-PAD conditions were: 100% A for 5 min, 0–12% B for 15 min, 12–100% B for 5 min, 100–0% B for 2 min, 100% A for 8 min, with a flow rate of 0.3 mL/min at 30 °C. A mixture of xylose (Sigma-Aldrich, St. Louis, USA) and oligosaccharides with 2 to 6 xylose residues (X2 to X6) (Megazyme, Wicklow, Republic of Ireland) was employed as chromatographic standards.

Next, enzymatic products from glucuronoxylan degradation were evaluated by matrix-assisted laser desorption/ionization with time-of-flight detection spectrometry (MALDI-TOF) using Microflex LT MALDI-TOF equipment (Bruker Daltonics, Massachusetts, EUA). One μL of a mixture containing a 1:1 ratio of the reaction products and 2,5-dihydroxybenzoic acid (DHB) (Sigma-Aldrich, St. Louis, USA) matrix, prepared as a 20 mg/mL stock in TA30 solvent (30:70 (v/v) acetonitrile:TFA 0.1% in water), was applied to three different spots of the MSP 96 polished steel target (Bruker Daltonics, Massachusetts, EUA). After drying, the spectrum of the samples was acquired using linear positive-ion reflector mode (6 laser shots average, in the 500–1500 *m*/*z* range). The results were analyzed using flexAnalysis software (Bruker Daltonics, Massachusetts, EUA) and Version 2020 of OriginLab.

### 2.5 Glucuronoxylanase Crystallization, Data Collection, and Analysis

*Bp*Xyn30_8A in a stock solution at 10 mg/mL in Tris-HCl (20 mM, pH 7.5) and 75 mM NaCl was screened using commercial crystallization kits in the sitting-drop vapor diffusion settings. Crystallization plates were stored in Rock Imager 1000 (Formulatrix, Bedford, MA, USA) equipment. Initial needle-shaped crystals were observed under conditions with 20% (w/v) PEG 3350 and 0.18 M ammonium citrate. Next, the crystallization conditions were optimized using the hanging-drop approach by altering the precipitant from 15 to 27.5% and salt from 50 to 300 mM in 24-well crystallization plate settings. Suitable crystals for diffraction experiments were obtained in 25% (w/v) PEG 3350 and 0.2 M ammonium citrate crystallization conditions. Obtained crystals were mounted on CryoLoops, flash-frozen, and stored in a Unipuck system for subsequent diffraction data collection. The synchrotron data collection was carried out at the Brazilian Synchrotron Light Laboratory MANACA beamline (CNPEM, Sirius, Campinas, SP, Brazil).^21^ X-ray diffraction data were collected under a cold nitrogen stream with a Pilatus 2M detector (Dectris, Baden, Switzerland) and an X-ray energy of 12.688 keV using a fine ϕ-slicing strategy.^22^ 3600 images were acquired and further processed with the XDSGUI program package (Version January 10, 2022 ^23^).

AlphaFold-predicted structure of *Bp*Xyn30_8A^24^ was used as a molecular replacement search model to determine the X-ray structure of the enzyme with the Phaser program,^25^ and the Autobuild program was used for the 3D model construction.^26^ The structure refinement was conducted in PHENIX-refine^27^ and manually adjusted in Coot^28^ (Supporting Information). The final crystallographic model was deposited to the Protein Data Bank (PDB) and received the accession code 9O5H.

Sequence alignment of *Bp*Xyn30_8A and other 17 published GH30_8 glucuronoxylanases included in the CAZy database (http://www.cazy.org) (Supporting Information) was conducted in the Molecular Evolutionary Genetics Analysis software Version 11 (MEGA11)^29^ using the ClustalW algorithm.^30^ The alignment was submitted to ESPript 3.0 ^31^ for depiction of secondary structure and to the Consurf server^32^ for identification of conserved regions within *Bp*Xyn30_8A structure. *Bp*Xyn30_8A was used as the query in both cases. Active site residues were inferred by superposing the glucuronoxylanase structure with *Dc*Xyn30A bound to MeGlcA^2^X3 (PDB entry 2Y24 ^33^) and *At*Xyn30A mutant (E225A) complexed with both xylobiose and xylotriose (PDB entry 5A6L^34^). Furthermore, GLYCAM-Web (https://glycam.org/cb/) was used for the construction of xylohexaose decorated with GlcA in order to analyze the subsites that allow glucuronic acid substitutions. Images of the structures were generated using the PyMOL Molecular Graphics System (Version 2.5.2, Schrödinger, LLC, New York, USA), and the PyMOL plugin APBS Electrostatics^35^ was employed for visualization of *Bp*Xyn30_8A electrostatic potentials.

### 2.6 Corn Cob and *Eucalyptus* Pretreatment, Chemical Characterization, and Enzymatic Degradation

Corn cobs (CCs), which were applied in our assays, were purchased from a local supermarket and milled with a knife mill until a mash of approximately 20 was reached. The *Eucalyptus* sawdust (E) was obtained from a local sawmill (Araraquara, SP, Brazil). The biomass humidity was determined using a Moisture Balance MOC 120H (Shimadzu, Kyoto, Japan). Next, the biomass was stored in plastic bags until further use. 1% (w/v) NaOH in water and 10% (w/v) of biomass (dry weight) were used for the alkaline pretreatment, which was conducted in an autoclave at 121 °C for a duration of 40 min. Vacuum filtration was applied to separate the solids from the liquid, and the solids were thoroughly washed with running water until the pH reached 7. The final washing step included deionized water. After that, the pretreated biomass was left to dry at 50 °C in the incubator.

Chemical characterization of untreated and alkali-pretreated *Eucalyptus* (E-IN and E-Alk, respectively) was conducted in triplicate, as previously reported.^36^ CC-IN and CC-Alk chemical compositions were obtained in a previous work.^36^

E-IN, E-Alk, structural carbohydrates, and soluble lignins, were obtained in a reaction with 72% H_2_SO_4_ for 7 min at 45 °C with constant stirring. A ratio of 2 g biomass (dry weight) to 15 mL sulfuric acid was used. The mixture was incubated in an autoclave for 30 min at 121 °C with diluted acid (4% H_2_SO_4_).^36^ The soluble fraction was analyzed by a high-performance liquid chromatography (HPLC) system equipped with the Aminex HPX-87H (300 × 7.8 mm) or Aminex HPX-87P (300 × 7.8 mm) columns (Bio-Rad, California, USA) in order to quantify acetic acid and simple sugars, respectively. Calibration curves for glucose, xylose, arabinose, and acetic acid were used. Five mM sulfuric acid was the eluent for the Aminex HPX-87H column, while the eluent for the Aminex HPX-87P column was deionized water, and both run conditions employed a 0.6 mL/min isocratic flow for 60 min. Furthermore, the absorption of a mixture of the hydrolyzate at 5% and 6.5 M NaOH at 2% was measured at 280 nm using a quartz cuvette in a spectrophotometer for evaluation of the soluble lignin fraction.

The insoluble solid fraction was filtered through quantitative ashless filter paper (Whatman, Kent, U.K.) and washed with 1 L of deionized water. The filter with the solid fraction was dried at 105 °C for 2 h, and insoluble lignin was quantified by weighing the sample. Next, the ash content was determined by burning the biomass in a muffle furnace (using heating steps of 1 h at 200 °C, 1 h at 400 °C, and, finally, 2 h at 800 °C).

The enzymatic hydrolysis of alkaline-pretreated *Eucalyptus* sawdust and corn cobs was performed using 5% (w/v) biomass (dry weight) and 0.1 mg of the enzyme in 20 mM sodium phosphate buffer, pH 6, in a final volume of 1 mL. The samples were measured in triplicate and maintained at 40 °C and 1000 rpm in a Thermomixer C (Eppendorf, Hamburg, Germany). Control reactions in the absence of the enzyme were also performed. Aliquots were retrieved after 6 and 24 h, and the reactions were stopped by keeping the reaction mixtures at 95 °C for 10 min. Next, samples were centrifuged at 17000 *g* for 5 min, and the supernatants were passed through a 0.22 μm CHROMAFIL Xtra PTFE-20/25 syringe filter (Macherey-Nagel, Düren, Germany) prior to injection in HPAEC-PAD. The oligosaccharide analysis was conducted as described in Section 2.4. Furthermore, the reaction products were quantified in terms of xylose equivalents by the DNS technique.

### 2.7 Enzymatic Production of Acid XOS from Commercial Glucuronoxylan and Assessment of Prebiotic Activity

The production of XOS was conducted as follows: 1% (w/v) of beechwood xylan was mixed with 0.5 μM *Bp*Xyn30_8A in 20 mM sodium phosphate buffer, pH 6, at 40 °C and 150 rpm. After 24 h, the reactions were inactivated at 95 °C for 10 min and filtered using the 5K Amicon Ultra Filter (Merck, Darmstadt, Germany). The flow-through was collected and left in an open container at 50 °C until all the water evaporated. The residual solid was resuspended in 2 mL of water and quantified using the DNS assay. In addition, the linear oligosaccharide composition of XOS was determined by HPAEC-PAD using a calibration standard containing xylose (Sigma-Aldrich, St. Louis, USA) and XOS with DP from 2 to 6 (Megazyme, Wicklow, Ireland).

In order to assess the prebiotic activity of XOS, *B. adolescentis* was purchased from a local drugstore and cultured statically at 37 °C in thioglycolate media (HiMedia, Mumbai, India) for 48 h. Probiotic bacteria at an OD_600_ = 0.1 were then incubated in a medium composed of 10 g/L casein, 5 g/L yeast extract, 5 g/L peptone, 5 g/L NaCl, 2 g/L K_2_HPO_4_, 0.2 g/L MgSO_4_·(7H_2_O), 0.05 g/L MnSO_4_·(1H_2_O), 0.5 g/L cysteine·(1H_2_O), 0.025% (w/v) resazurin at pH 7.2, with or without 1 g/L carbon source (XOS produced by *Bp*Xyn30_8A or glucose). The carbon source was sterilized by filtration using 0.2 μm Millex PTFE sterile syringe filters (Merck, Darmstadt, Germany) prior to the assay and added last to the reaction. The samples were prepared in triplicate and kept in 5 mL glass vials at 37 °C under static conditions for up to 24 h. Two control samples were prepared: a sterile control (lacking both bacteria and XOS) and a second containing bacteria but no XOS. To account for the background contribution from bacterial growth supported by the medium, values from the bacterial control were subtracted from those of the XOS-supplemented samples.

After 4 and 24 h, aliquots were removed to measure the bacterial growth at OD_600_, and the rest of the solution was submitted to centrifugation at 13000 *g* for 5 min. The supernatant was used to determine the pH of the medium and the concentration of reducing sugar in terms of xylose using the DNS assay. Furthermore, glucose and short-chain fatty acids (SCFAs) were quantified in the fermented samples by HPLC using the Aminex HPX-87H (300 × 7.8 mm) column (eluent and run conditions as described in Section 2.6) and a standard curve containing glucose or a mixture of formic acid, acetic acid, propanoic acid, lactic acid, and butyric acid, respectively. The products were separated by chromatography using a refractive index detector.

## 3 Results and Discussion

### 3.1 Heterologous Expression and Purification of *Bp*Xyn30_8A

*Bp*Xyn30_8A gene was successfully amplified from the genomic DNA of *B. pumilus*, cloned into the pETTRXA-1a/LIC expression vector, and transformed into *E. coli* Rosetta (DE3) cells. The glucuronoxylanase was produced in a soluble form, and two purification steps using Ni-NTA resin were required: the first to separate the xylanase from host proteins and the second to remove the 6xHis-thioredoxin tag following TEV protease cleavage. *Bp*Xyn30_8A appeared as a single band on SDS-PAGE, with a molecular mass compatible with the theoretical molecular mass calculated based on its amino acid sequence (44.5 kDa) (Figure S1). The yield of the purified recombinant enzyme was 15 mg of protein per liter of culture.

### 3.2 Enzymatic Assays

#### 3.2.1 Optimum pH and Temperature

Optimal conditions for *Bp*Xyn30_8A enzymatic assays were assessed by varying either the pH or temperature of the reactions (Figure S2a and b). *Bp*Xyn30_8A exhibited the highest activity at pH 7, whereas its activity at pH 6 and 8 was 90% and 84% of its maximum activity, respectively. The optimum temperature tests revealed that the glucuronoxylanase displays the best activity between 50 and 60 °C. Prokaryotic glucuronoxylanases from family 30 have been described in the BRENDA database (https://www.brenda-enzymes.org/index.php)^37^ as presenting a broad range of optimum pH and temperature, i.e., with pHs from 5 to 10 and temperatures between 30 and 70 °C, although most of them function best at neutral pH and mild temperatures (40 °C).^18^ DSF assays are consistent with the enzymatic evaluation of optimum pH and temperature: the glucuronoxylanase was more stable in buffers with pHs from 5 to 8 (with melting temperatures between 60 and 64 °C; see Figure S2c).

#### 3.2.2 Residual Activity Assays of BpXyn30_8A

*Bp*Xyn30_8A thermostability was evaluated in order to determine appropriate temperatures for XOS production. The enzyme is very stable at 50 °C, with a half-life of 36 ± 2 h. However, at 55 °C it is rapidly inactivated (*t*_1/2_ = 3.6 ± 0.1 h) (Figure S3). In contrast, *Rc*Xyn30A used as a means of comparison in further experiments, performs better at 40 °C, with a half-life of 45 ± 6 h.^18^ Since both enzymes have long half-lives at lower temperatures, we decided to conduct the enzymatic XOS production experiments from pretreated biomass and glucuronoxylan at 40 °C.

Next, *Bp*Xyn30_8A resistance to salt and ethanol was assessed (Figure 1). The glucuronoxylanase displayed high resistance to salt (Figure 1a), maintaining its activity after incubation with 2.8 M of NaCl. This result is in line with the literature, since most salt-tolerant glucuronoxylanases have been isolated from *Bacillus* strains.^38^ Furthermore, *Bp*Xyn30_8A uphold over 80% and 50% of its activity after 1 h in a solution containing 5% or 20% (v/v) ethanol (Figure 1b). Halotolerance and ethanol tolerance are desirable characteristics for enzyme candidates used in processes that employ high concentrations of salt and ethanol, such as in the food, beverage, and biofuel industries. For instance, commercial enzymatic preparations containing xylanases have been used for winemaking^39^ and brewing soy sauce.^38^

**Figure 1:**
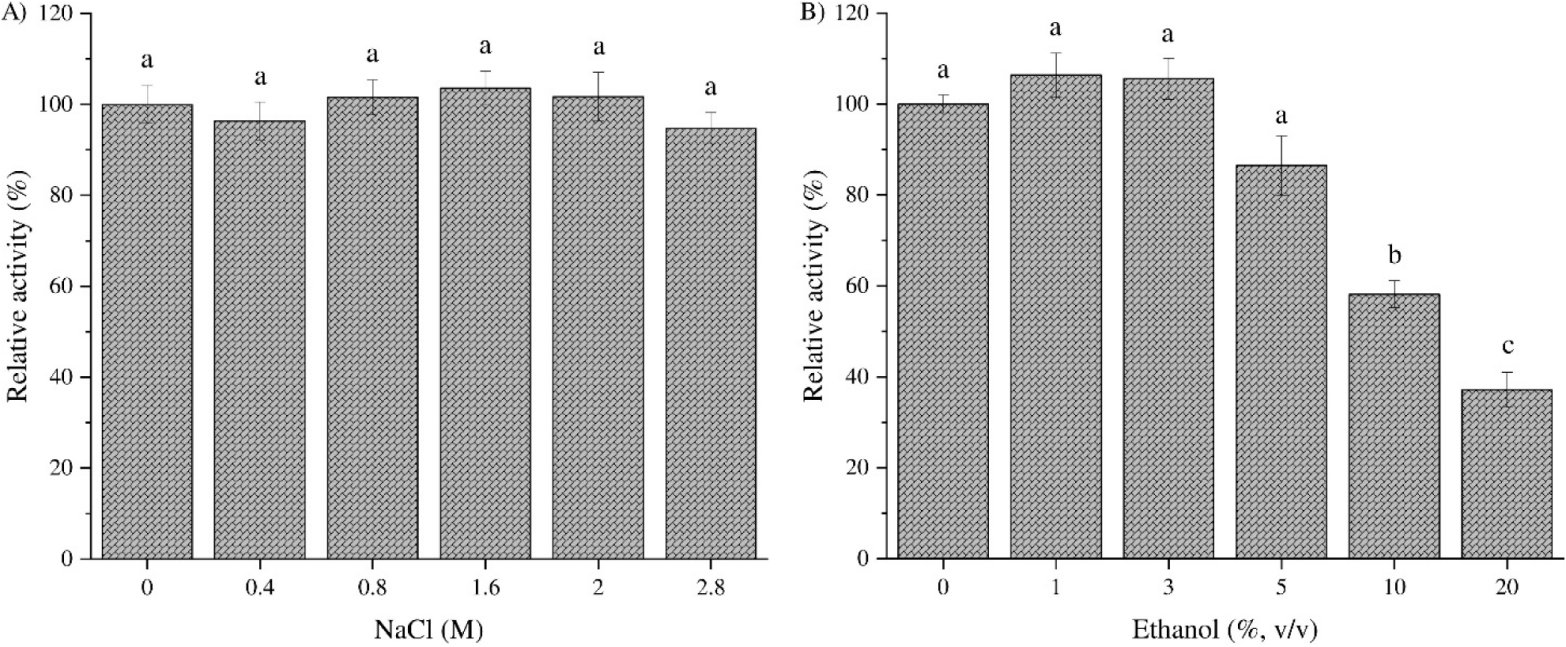
Enzymatic activity of *Bp*Xyn30_8A in increasing concentrations of (a) NaCl and (b) ethanol. The glucuronoxylanase was incubated for 1 h at room temperature with salt or ethanol in 20 mM Tris-HCl buffer, pH 7, and aliquots were removed and used for DNS assays. The data presented were measured in triplicate (*n* = 3), and error bars are ±SD. ANOVA and Tukey test were used for mean comparison, and different letters refer to statistically different values (*p* < 0.05).

#### 3.2.3 The Enzyme Cleavage Pattern

The profile of the hydrolytic products released by *Bp*Xyn30_8A upon the hydrolysis of beechwood glucuronoxylan was evaluated as a function of time by using HPAEC-PAD (Figure 2a). In contrast to most representatives of this family, such as *Bs*Xyn30A,^40^ *Dc*Xyn30A,^41^ and *Rc*Xyn30A,^18^ which liberate mainly monoglucuronylated xylooligosaccharides as the hydrolytic products, significant amounts of xylobiose, xylotriose, xylotetraose, xylopentaose, and xylohexaose were observed in *Bp*Xyn30_8A xylan hydrolysis. The amount of linear XOS increased over time, which suggests that the glucuronoxylanase is able to recognize and cleave linear xylan sites, i.e., without MeGlcA ramification. In accordance with the HPAEC-PAD results, the 24-h sample analyzed by MALDI-TOF (Figure 2b) contained linear xylooligosaccharides along with MeGlcA-branched XOS (DP: 3–7).

**Figure 2:**
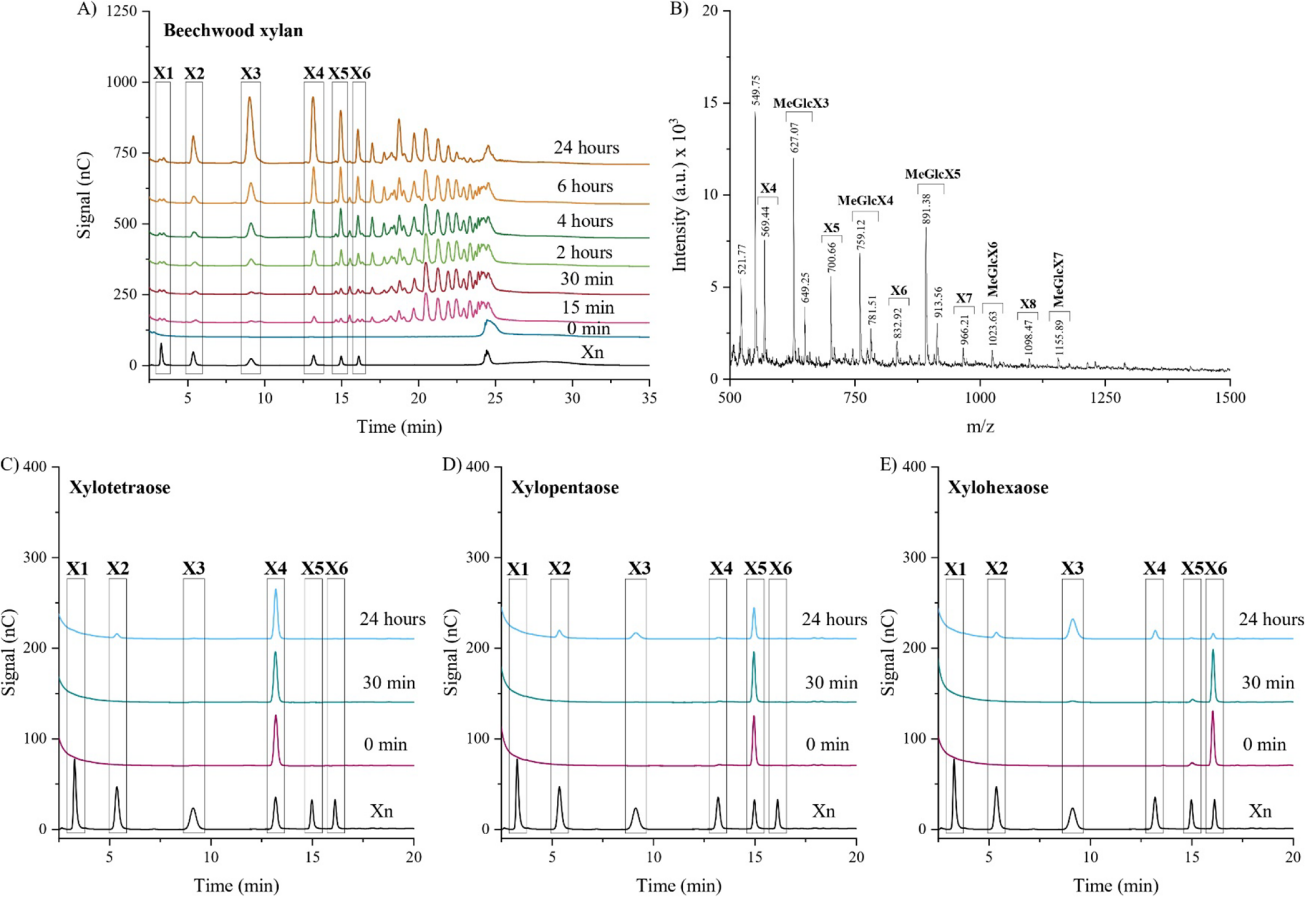
Enzymatic cleavage pattern of *Bp*Xyn30_8A of (a, b) 0.5% (w/v) beechwood glucuronoxylan (upper panels) or 0.05 mg/mL of (c) xylotetraose, (d) xylopentaose, or (e) xylohexaose (lower panels). The reactions were conducted by using 180 nM *Bp*Xyn30_8A in Tris-HCl buffer (20 mM, pH 7) and maintained at 50 °C for up to 24 h. The samples were analyzed by HPAEC-PAD (a, c, d, e) or MALDI-TOF MS (b). The standards are Xn: xylose and xylooligosaccharides (DP: 2–6), X3: xylotetraose, X4: xylotetraose, X5: xylopentaose, X6: xylohexaose. The peaks in the MALDI-TOF spectrum were attributed as the masses of sodium adducts of XOS and aldouronic acids.

In order to investigate the decoration requirement of *Bp*Xyn30_8A, the enzyme was incubated with xylotetraose (Figure 2c), xylopentaose (Figure 2d), and xylohexaose (Figure 2e). Hardly any cleavage product was observed after 30 min of reaction, but xylohexaose was slowly converted to X2, X3, and X4, and xylopentaose was slowly converted to X2 and X3 after 24 h. In addition, subtle amounts of X2 could be detected when xylotetraose was used as a substrate. Similar patterns were observed upon incubation of the enzyme with 10-fold higher amounts of X4–X6 (Figure S4). Šuchová et al. (2018)^41^ highlighted the importance of MeGlcA ramifications for substrate recognition by GH30_8 glucuronoxylanases. For instance, the enzyme studied by the group (*Dc*Xyn30A) exhibited a much higher specific activity toward branched XOS compared to unbranched ones.

All in all, *Bp*Xyn30_8A can cleave unbranched substrates, albeit at a slower rate, which contributes to the production of short branched and unbranched oligosaccharides by the enzyme. In contrast, the other member of the GH30_8 family, also used in the present work as a means of comparison (*Rc*Xyn30A), liberates mainly long branched XOS (DP: 5–12).^18^

#### 3.2.4 Specific Activity and Kinetic Parameters of the Enzyme

*Bp*Xyn30_8A activity was evaluated against eight potential substrates, including arabinoxylan, and, consistent with the predominant activity of glucuronoxylanases from the GH30_8 subfamily,^7^ it exhibited catalytic activity exclusively on glucuronoxylan. The enzyme presented a specific activity of 13.5 ± 0.7 U/mg when beechwood 4-O-methyl-glucuronoxylan was used as a substrate. The latter substrate contains ∼13% of 4-O-methyl glucuronic acid chemically attached by α-1,2 bonds to the xylopyranose backbone, according to the manufacturer. *Bp*Xyn30_8A has one of the lowest specific activities among the characterized GH30 glucuronoxylanases, including *Rc*Xyn30A, which displayed a specific activity of 125.6 ± 5.4 U/mg.^18^

Next, the kinetic parameters of *Bp*Xyn30_8A and *Rc*Xyn30A were determined using beechwood glucuronoxylan as a substrate (Table 1). Table 1 also contains the kinetic parameters of other published GH30_8 glucuronoxylanases. *Bp*Xyn30_8A binds more strongly to beechwood glucuronoxylan compared to *Rc*Xyn30A, as manifested by their *K_M_*. This may indicate that *Rc*Xyn30A has a higher affinity for the glucuronic acid substitutions, which could interfere with the enzyme binding to the substrate. At the same time, *Bp*Xyn30_8A might bind to other regions of glucuronoxylan, including the linear ones. On the other hand, the *Rc*Xyn30A catalytic efficiency (*k*_cat_/*K_M_*) is almost 3 times higher than the catalytic efficiency of *Bp*Xyn30_8A. The values obtained fall within the range of kinetic parameters reported for previously characterized glucuronoxylanases from the same family (Table 1).

**Table 1:** Kinetic Parameters of *Bp*Xyn30_8A, *Rc*Xyn30A, and Other Glucuronoxylanases from the GH30_8 Family

Organism/glucuronoxylanase	Substrate	*K*_*M*_ (mg·mL^–1^)	*k*_cat_ (s^–1^)	*k*_cat_/*K*_*M*_ (mL·mg^–1^·s^–1^)	Reference
*Bacillus pumilus*/*Bp*Xyn30_8A	Beechwood xylan	4.8 ± 0.3	26.7 ± 0.5	5.5 ± 0.4	Present work
*Ruminococcus champanellensis* /*Rc*Xyn30A full	Beechwood xylan	18.4 ± 2.2	343.7 ± 24.8	18.7 ± 3.6	Present work
*Cellulosilyticum ruminicola* MMBC-1/*Cr*Xyn30B	Glucuronoxylan	0.7 ± 0.0	11.8 ± 0.4	16.9 ± 0.8	^42^
*Melioribacter roseus* P3M-2*/Mr*Xyn30A	Birchwood xylan	10.5 ± 3.0	104.4±^a^	9.9±^a^	^43^
*Bacillus subtilis* *str.* 168*/Bs*Xyn30A	Sweetgum xylan	1.63±^a^	2.64±^a^	1.62±^a^	^40^
*Clostridium acetobutylicum* ATCC 824/*Ca*Xyn30A	Beechwood xylan	3.12 ± 0.15	86 ± 8	27.6 ± 3.9	^44^
*Dickeya chrysanthemi* D1*/Dc*Xyn30A	Glucuronoxylan	1.64 ± 0.42	34.1 ± 1.6	20.8 ± 5.4	^41^
*Acetivibrio thermocellus* ATCC 27405*/At*Xyn30A	Beechwood xylan	2.2±^a^	2008.3±^a^	912.9±^a^	^45^
*Paenibacillus favisporus* CC02-N2*/Pf*Xyn30A	Beechwood xylan	4±^a^	222±^a^	55±^a^	^46^
*Paenibacillus barcinonensis* BP-23*/Pb*Xyn30A	Beechwood xylan	14.72±^a^	25.17±^a^	1.71±^a^	^47^

a Experimental errors have not been reported.

### 3.3 X-ray Structure of BpXyn30_8A

The crystal structure of *Bp*Xyn30_8A was determined to have a 2.16 Å resolution. Data collection and refinement statistical parameters are summarized in Table S2. *Bp*Xyn30_8A crystallographic structure has a typical (β/α)_8_ TIM barrel fold that harbors the substrate cleavage site and a β-sheet immunoglobulin-like domain appended to the barrel (Figure 3a). The importance of the 9 β-strands was demonstrated in a 2012 work,^47^ in which the segments of the β-structure were removed from the full-length *P. barcionensis* GH30_8 glucuronoxylanase. All truncated enzymes showed no hydrolytic activity against xylan, demonstrating that the full-length domain is necessary for the enzymatic activity. In addition, the crystal structure of a *B. subtilis* glucuronoxylanase^48^ revealed that the β_9_-domain binds to MeGlcAX2, suggesting its possible involvement as a putative CBM.

**Figure 3:**
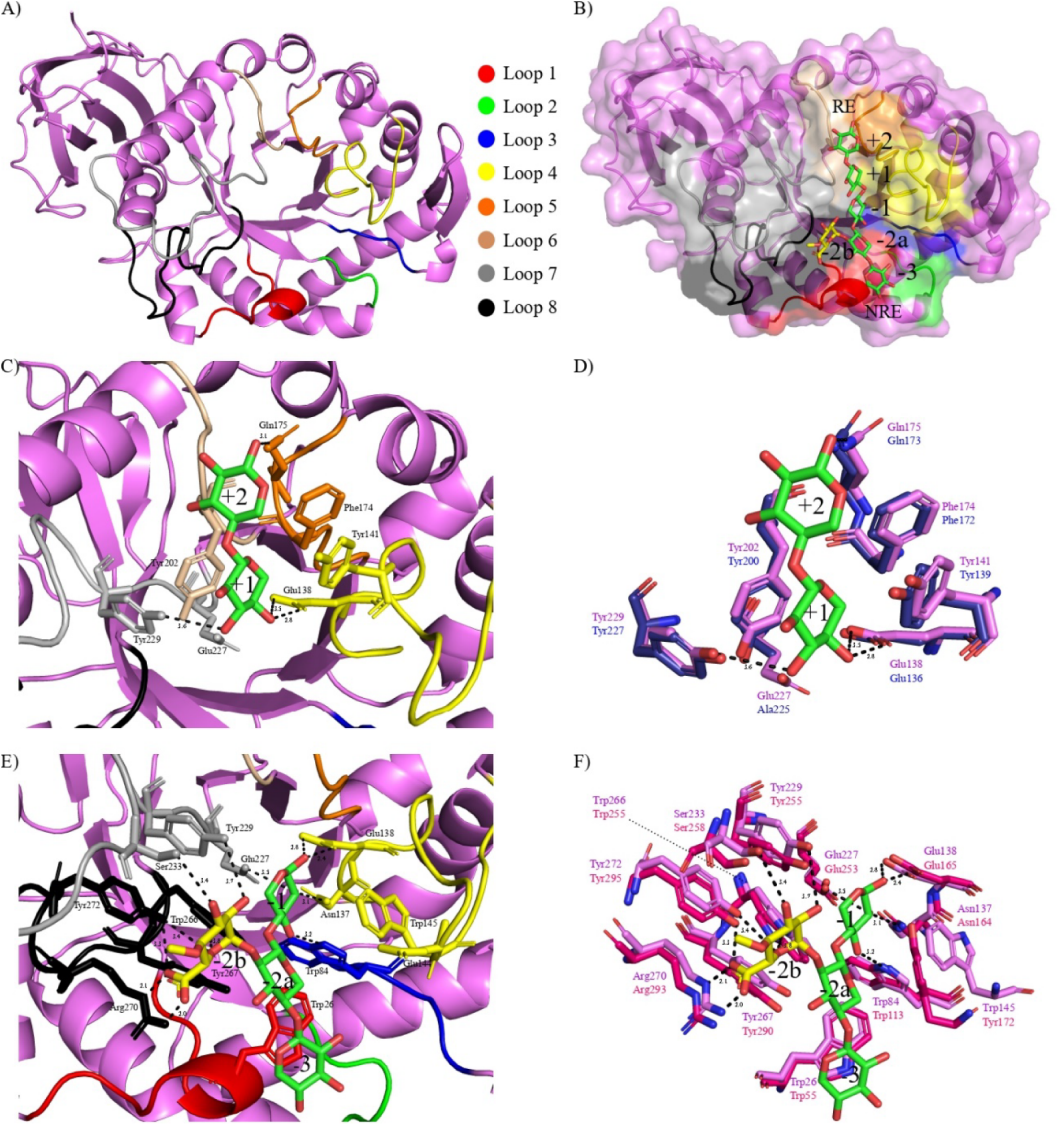
Crystallographic structure and structural bases for decorated substrate recognition by *Bp*Xyn30_8A. (a) Front view of the enzyme depicting the loops that surround the catalytic site. Loops were numbered and colored according to Vacilotto et al. (2024),^18^ as follows: L1 in red (loop β1-α1), L2 in green (loop β2-α2), L3 in blue (loop β3-α3), L4 in yellow (loop β4-α4), L5 in orange (loop β5-α5), L6 in salmon (loop β6-α6), L7 in gray (loop β7-α7), and L8 in black (loop β8-α8). (b) Cartoon and surface representation of *Bp*Xyn30_8A in the presence of MeGlcA^4^X5. The ligand was obtained by superposing the glucuronoxylanase structure with *Dc*Xyn30A bound to MeGlcA^2^X3 (PDB entry 2Y24) and *At*Xyn30A complexed with xylobiose (PDB entry 5A6L). RE and NRE denote reducing end and nonreducing ends. Positioning of (c, d) xylobiose and (e, f) MeGlcA^2^X3 in *Bp*Xyn30_8A aglycone and glycone sites, respectively, and residues involved in substrate recognition. Amino acids in the vicinity of the substrate binding cleft of *Bp*Xyn30_8A are shown as violet sticks, and a comparison was conducted with either (d) *At*Xyn30A (in dark blue) or (f) *Dc*Xyn30A (in pink) residues. Interactions with the ligands are shown by black dashed lines, and distances are shown in Å.

Out of the eight β-α loop regions surrounding the catalytic substrate binding site of *Bp*Xyn30_8A (Figure 3a and b), only the loop L2 does not interact with the xylosyl chain. Sequence alignment with studied GH30_8 enzymes (Figure S5) allowed us to identify 17 amino acids within the seven loops that form *Bp*Xyn30_8A subsites and might interact with the substrate, including the catalytic residues (Glu138 and Glu227). To further analyze interactions in the aglycone subsites, we superposed *Bp*Xyn30_8A structure with the mutant *At*Xyn30A-E225A bound to xylobiose (PDB entry 5A6M,^34^ Figure 3c and d), whereas glycone subsites were assessed mainly by superimposition with *Dc*Xyn30A bound to MeGlcA^2^X3 (PDB entry 2Y24 ^33^, Figure 3e and f), which have RMSDs of 0.368 Å and 0.792 Å, respectively. All interactions between *Bp*Xyn30_8A amino acids and MeGlcA^4^X5 built with ligands of the structures mentioned above, are detailed in Table 2.

**Table 2:** Possible Interactions between *Bp*Xyn30_8A Amino Acids and MeGlcA^4^X5

Subsite	Amino acid/atom	Sugar/atom	Type of interaction	Distance, Å
+2	Phe174	Xyl*p*	Stacking	5.9
+2	Gln175/NE2	Xyl*p*/O1	Hydrogen bond	3.1
+1	Tyr141	Xyl*p*	Stacking	5.2
+1	Tyr202	Xyl*p*	Stacking	4.2
+1	Tyr229/OH	Xyl*p*/O3	Hydrogen bond	3.6
+1	Glu138/OE1	Xyl*p*/O4	Hydrogen bond	2.8
+1	Glu138/OE2	Xyl*p*/O4	Hydrogen bond	3.5
–1	Glu138/OE1	Xyl*p*/O1	Hydrogen bond	2.4
–1	Glu138/OE2	Xyl*p*/O1	Hydrogen bond	2.8
–1	Glu227/OE2	Xyl*p*/O2	Hydrogen bond	3.5
–1	Asn137/ND2	Xyl*p*/O2	Hydrogen bond	3.1
–1	Trp84/NE1	Xyl*p*/O3	Hydrogen bond	3.2
–1	Trp266	Xyl*p*	Stacking	4.8
–1	Trp145	Xyl*p*	Stacking	6.0
–2a	Trp26	Xyl*p*	Stacking	4.7
–2a	Tyr267	Xyl*p*	Stacking	6.2
–2b	Arg270/NH2	MeGlcA/O6B	Ionic interaction	2.0
–2b	Arg270/NE	MeGlcA/O6A	Ionic interaction	2.1
–2b	Tyr229/OH	MeGlcA/O2	Hydrogen bond	3.7
–2b	Tyr272/OH	MeGlcA/O5	Hydrogen bond	3.4
–2b	Tyr272/OH	MeGlcA/O6A	Hydrogen bond	3.3
–2b	Trp266/NE1	MeGlcA/O5	Hydrogen bond	3.8
–2b	Ser233/OG	MeGlcA/O3	Hydrogen bond	3.4
–3	Trp26	Xyl*p*	Stacking	3.9

Among the amino acid residues involved in substrate recognition, the ones responsible for the accommodation of MeGlcA in the often-called subsite −2b have been shown to be essential for glucuronoxylan binding. In addition to five hydrogen bonds, the sugar is also coordinated by two ionic interactions between the MeGlcA carboxylate group and the arginine residue guanidine group (Arg270 in *Bp*Xyn30_8A). The binding energy of MeGlcA by *Dc*Xyn30A, for instance, was estimated to correspond to 36% of the total energy required for the binding of MeGlcA^2^X3.^33^ Not surprisingly, enzymes that behave as nonspecific glucuronoxylanases have another amino acid in place of arginine. *Ruminiclostridium papyrosolvens* GH30_8 enzyme,^49^ for example, has a tryptophan substitution, which implies activity of *Rp*Xyn30A against arabinoxylan. Therefore, one can conclude that the cleavage products of glucuronoxylan by *Bp*Xyn30_8A most likely present the glucuronic acid bound to the last but one Xyl*p* residue at the nonreducing end, as reported previously.^50^

The binding site of *Bp*Xyn30_8A contains two positive and three negative subsites (Figure 3b). Xylose residues positioned in subsites −1 and +1 are stabilized by a network of hydrogen bonds and two stacking interactions. In contrast, in the remaining subsites, the Xyl*p* units are held mainly by hydrophobic interactions. Analysis of amino acid residues in the vicinity of the binding site shows a high degree of sequence conservation, which is more pronounced in the region spanning subsites −1 to +2 (Figure S6). Additionally, the residues of *Bp*Xyn30_8A exhibit a net negative charge at the binding site of the xylose chain, with a slightly negative potential near the MeGlcA decoration subsite.

We also evaluated the subsites, in addition to subsite −2 of *Bp*Xyn30_8A, that could accommodate decorations by manually inserting a GlcA group at the O2 position of Xyl*p* residues of MeGlcA^4^X5 (Supporting data) to analyze potential steric hindrance in the active site of *Bp*Xyn30_8A. As observed for *Rc*Xyn30A (PDB entry 8VG9 ^18^), the O2 atom of xylopyranose moieties located at subsites +2 and −3 is oriented outward from the enzyme surface, suggesting that these positions may tolerate decorations. The structural similarities between the two enzymes extend beyond this observation, where the substrate-binding site residues in *Bp*Xyn30_8A are also conserved in *Rc*Xyn30A.^18^ A notable difference is the presence of a tryptophan residue in *Rc*Xyn30A that forms an additional +3 subsite, which is absent in *Bp*Xyn30_8A.

Since the ligand interaction with the amino acid residues of *Bp*Xyn30_8A and *Rc*Xyn30A does not fully explain the differences in cleavage patterns observed between these enzymes, a comparative analysis of their binding clefts was conducted to identify potential structural determinants underlying this divergence. A 3D comparison with other prokaryotic GH30 glucuronoxylanase X-ray structures available in the PDB (Figure 4a) revealed that several enzymes, such as *Bp*Xyn30_8A, *Bs*Xyn30A (PDB entry 3KL0 ^48^), and *At*Xyn30A (PDB entry 4CKQ), exhibit a β-sheet motif followed by loop 3 (β3-α3, demarcated by a rectangle). In contrast, both *Rc*Xyn30A and *Dc*Xyn30A have loops in this region. In *Bs*Xyn30A, this part of the structure is stabilized by an arginine side chain from loop 3 via stacking interaction with a tryptophan residue from loop 4, and a hydrogen bond between the carbonyl of the main chain of the lysine residue and a main chain nitrogen of the tryptophan.^48^ These interactions were also detected in *Bp*Xyn30_8A structure, occurring between Arg96, Trp147, and Lys102 residues (Figure 4b).

**Figure 4:**
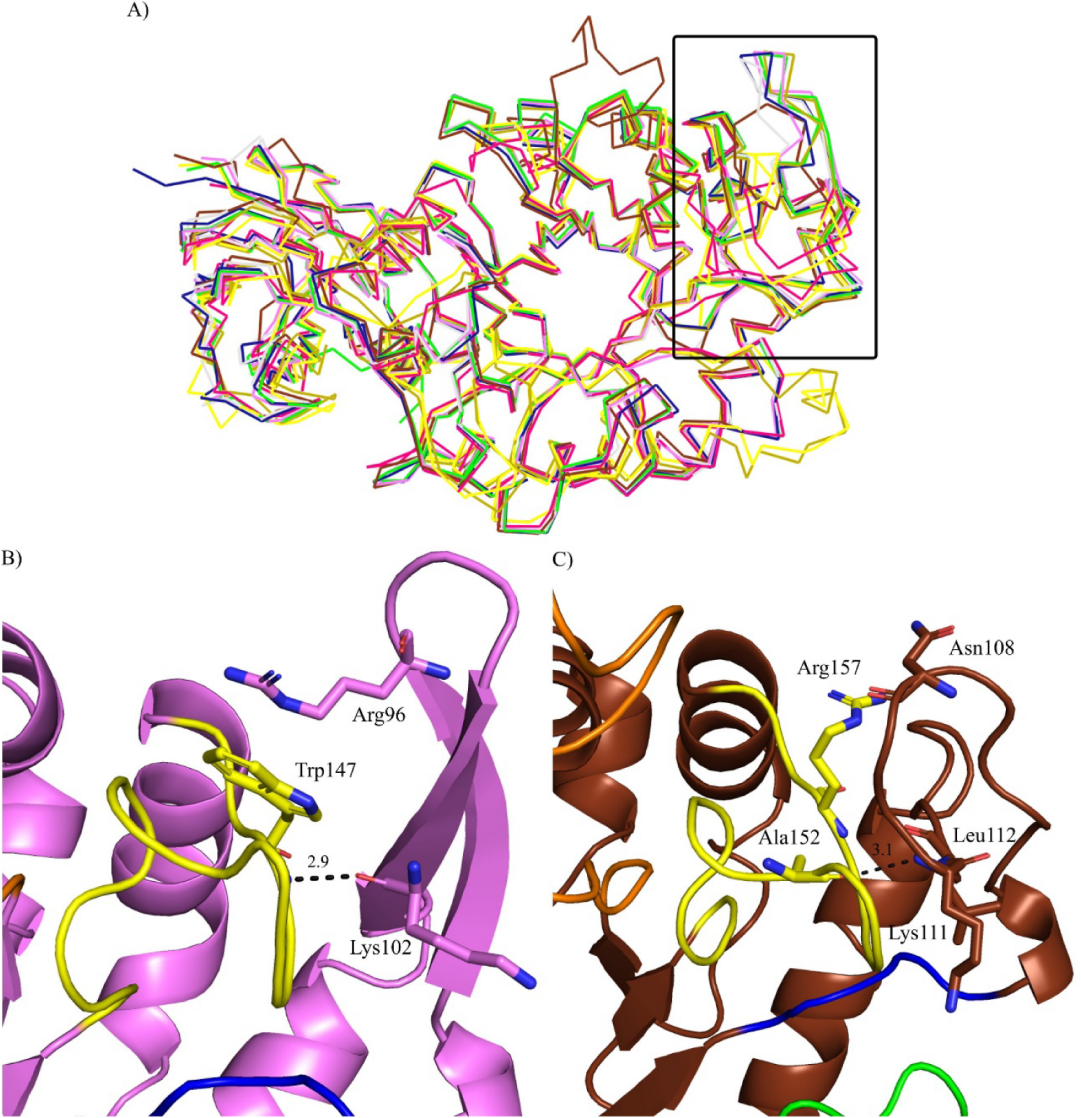
Structural superposition of glucuronoxylanases studied here with other crystallographic structures of prokaryotic GH30 available in the PDB. (a) Ribbon representation of superimposed *Bp*Xyn30_8A (violet), *Rc*Xyn30A (brown, PDB entry 8VG9), *Dc*Xyn30A (pink, PDB entry 2Y24), *At*Xyn30A (dark blue, PDB entry 4CKQ), *Bs*Xyn30A (green, PDB entry 3KL0), *Ca*Xyn30A (yellow, PDB entry 5CXP), *Rp*Xyn30A (olive green, PDB entry 4FMV), *Pb*Xyn30A (light gray, PDB entry 4QAW). The region highlighted with a rectangle in panel (a) shows the presence or absence of a β-fold after loop β3-α3. Cartoon representation of amino acid interactions between loop 4 (yellow, β4-α4) and the region after loop 3 (blue, β3-α3) of (b) *Bp*Xyn30_8A and (c) *Rc*Xyn30A. Hydrogen bonds are shown with black dashed lines, and all distances are in Å.

For the *Rc*Xyn30A enzyme, the only stabilizing interaction observed was a hydrogen bond between the Lys111 amino group, which is in a similar structural position to Lys102 of *Bp*Xyn30_8A, and the carboxyl atom of Ala152 (Figure 4c). No additional interactions that could contribute to the stabilization of this region were identified. The more extensive interaction network present in *Bp*Xyn30_8A suggests that the β4−α4 region may play a role in substrate accommodation and the cleavage pattern. However, this hypothesis remains to be validated experimentally. Mutational analyses of the residues involved in loop 3–loop 4 interactions would be necessary to establish a relationship between these structural features and the observed enzymatic specificity.

### 3.4 Enzymatic Hydrolysis of Alkaline-Pretreated Biomass Using Two Different Glucuronoxylanases

First, we set out to determine the composition of both untreated biomasses and the biomasses pretreated with 1% (w/v) NaOH (Figure 5a). The xylan of E-IN and CC-IN contained 52% and 11% acetylation, respectively, while the latter further contained ∼7% arabinofuranosyl units. As expected, alkaline pretreatment increased the fractions of cellulose and hemicellulose, and given that the xylan fraction in CC-Alk was approximately 3.5 times higher than in E-Alk, the CC-Alk seemed to be more promising for enzymatic hydrolysis, despite the presence of arabinose decorations, which could restrict the substrate cleavage sites and its accessibility.

**Figure 5:**
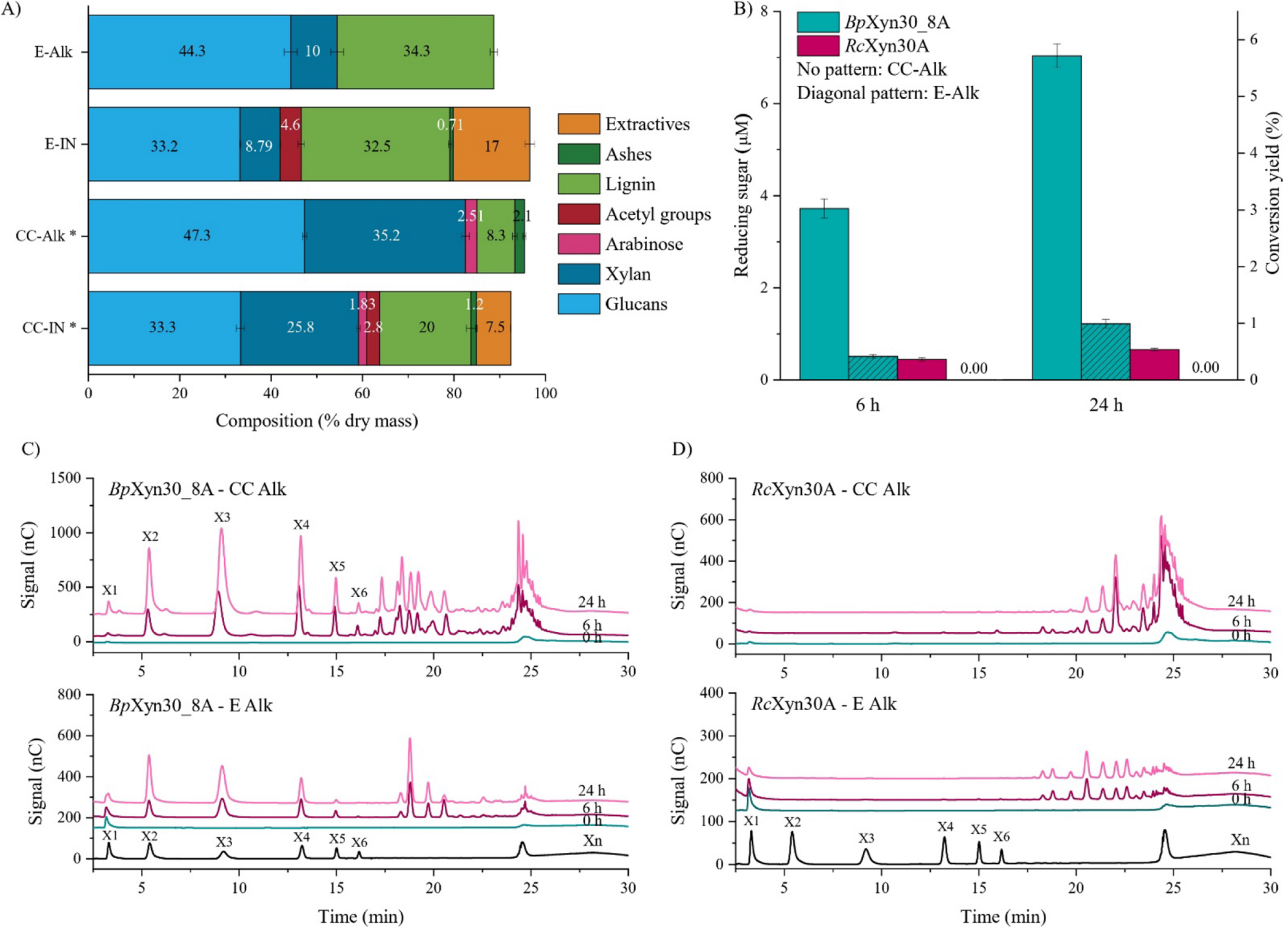
Enzymatic hydrolysis of biomass pretreated with 1% (w/v) NaOH. (a) Corn cob (CC) and *Eucalyptus* sawdust (E) composition as a % of dry mass prior to (CC-IN and E-IN) and after alkali pretreatment (CC-Alk and E-Alk). *CC-IN and CC-Alk chemical compositions were obtained previously.^36^ (b) Conversion yield of CC-Alk and E-Alk by *Bp*Xyn30_8A and *Rc*Xyn30A as quantified by reducing sugar. The DNS method did not allow detection of product formation in *Rc*Xyn30A degradation of E-Alk. HPAEC-PAD chromatograms of (c) *Bp*Xyn30_8A and (d) *Rc*Xyn30A hydrolyzes of the pretreated biomasses for 6 and 24 h. The enzymatic hydrolysis was conducted using 5% (w/v) biomass (dry weight) and 0.1 mg of protein in phosphate buffer (20 mM, pH 6) at 40 °C. Data presented are the mean value of triplicates (*n* = 3), and the error bars are ±SD.

The untreated *Eucalyptus* sawdust (E-IN) chemical composition is similar to previously reported compositions of *Eucalyptus* woodchips,^51^ *E. grandis*,^52,53^ *E. grandis* × *E. urophylla*,^53^ and a mixture (1:1) of *E. grandis* and *E. urophylla*.^54^ Reported values are 39–44% glucans, 14–27% lignin, 9–36% hemicellulose, 0.2–7% ashes, 3–27% extractives, and ∼ 3% acetyl groups. The highest fraction of lignin was reported for *E. grandis* sawdust and the mixture of *E. grandis* and *E. urophylla*, whereas *E. grandis* and *E. grandis* × *E. urophylla* bark showed the largest quantity of extractives. Xylan was highest in *Eucalyptus* woodchips and lowest in *E. grandis* bark.

While the alkaline pretreatment of corn cobs resulted in their considerable delignification, with a reduction of ∼12% in lignin dry mass, it practically had no effect on the delignification of *Eucalyptus* sawdust but led to xylan deacetylation. The same pattern was previously observed with *E. grandis* × *E. urophylla* woodchips treated with 4% (w/v) NaOH at 90 °C for 2 h.^55^ Although this pretreatment only slightly reduced the lignin fraction (by less than 3%), it resulted in the opening of the biomass cell wall structure and an increase in the surface area of the fibers.^55^ Therefore, we attributed the delignification difference to the differences in the physical and chemical structures of corn cobs and *Eucalyptus* residues.

Next, we proceeded by enzymatically treating the alkaline-pretreated biomasses with either *B. pumilus* or *R. champanellensis* glucuronoxylanases. CC-Alk and E-Alk enzymatic hydrolysis demonstrated that both *Bp*Xyn30_8A and *Rc*Xyn30A were capable of hydrolyzing the respective xylan fractions, which is consistent with the presence of (methyl)­glucuronic acid decorations (Figure 5). Surprisingly, quantification of reducing sugars showed that although *Rc*Xyn30A has a much higher specific activity against glucuronoxylan than *Bp*Xyn30_8A, the latter liberated 8 and 10 times more soluble products after being incubated with CC-Alk for 6 and 24 h, respectively (Figure 5b).

This difference might be explained by taking into account *Rc*Xyn30A affinity for glucuronic acid (GlcA) decorations, as alluded to in Section 3.2.4. In addition to this substrate specificity, other factors likely contribute to its lower yield on pretreated biomass. One such factor is the potential enzyme inhibition by biomass-derived compounds, such as phenolic compounds, furans, and organic acids.^56^ Furthermore, unproductive adsorption of the enzyme onto lignin or other biomass components would decrease its availability for xylan hydrolysis.^57^

DNS assay did not reveal XOS released by *Rc*Xyn30A from E-Alk, likely because of the small xylan fraction in the samples and the limited sensitivity of the DNS method. At the same time, HPAEC analysis demonstrated that substrate hydrolysis occurred, and all the obtained chromatograms were consistent with those obtained for glucuronoxylanase hydrolysis of beechwood xylan (Figure 5c and d).^18^ To sum up, as initially expected, the XOS yield from CC-Alk was considerably higher than that from E-Alk, showing that the high percentage of xylan in the biomass is more important than the presence of only one type of decoration. In addition, the amounts of XOS products generated by both glucuronoxylanases are time-dependent, which potentially allows for the optimization of XOS yields.

From an application perspective, GH30_8 xylanases, such as *Bp*Xyn30_8A, differ significantly from more widely studied xylanases, particularly those from families GH10 and GH11. GH10 xylanases are characterized by their broad substrate specificity, due in part to their more open active site that accommodates various substitutions, including arabinose and glucuronic acid side chains. This makes this family well-suited for the hydrolysis of highly decorated xylans from plant biomass. GH11 xylanases, in contrast, have a narrow active site and typically prefer linear, unsubstituted regions of the xylan backbone, as they are less permissive in terms of substrate decorations.^58,59^

GH30_8 enzymes, while less common, exhibit high specificity for glucuronoxylan and are particularly valuable for the targeted production of acidic XOS, also referred to as glucuronoxylooligosaccharides (GXOS). These compounds have attracted interest due to their potential as functional prebiotics and antioxidants.^60,61^ Their strict substrate requirements, however, limit their applicability in industrial biomass processing, where GH10 and GH11 enzymes, with their broader substrate specificity and higher catalytic efficiencies, remain the preferred options.

### 3.5 Prebiotic Activity of XOS Produced by *Bp*Xyn30_8A

In order to assess whether acidic XOS can be consumed by probiotic bacteria *B. adolescentis*, the oligosaccharides were produced using commercial glucuronoxylan and the glucuronoxylanase *Bp*Xyn30_8A, which yielded both linear and decorated XOS with a MeGlcA on the penultimate xylose moiety from the reducing end. As a means of comparison, bacteria were supplemented with the same amount of glucose in a separate experiment.

*B. adolescentis* was able to proliferate in medium containing either glucose or the enzymatically produced XOS, as can be observed by the increase in OD_600_ Microorganism population reached a stationary phase after 4 h of incubation with glucose (OD_600_ = 0.171), whereas it increased 250-fold when kept for 24 h in medium containing XOS when compared to the 4 h of fermentation (Figure 6a, upper panel). The stationary phase was probably caused by the depletion of glucose, which was completely consumed in the first hours of reaction (Figure 6a, lower panel). In contrast, only approximately 65% of the XOS available in the medium was used by the bacteria after 24 h (Figure 6a, lower panel). Moreover, sugar fermentation by *B. adolescentis* resulted in a decrease in the pH of the samples (Figure 6a, middle panel), which was more expressive when XOS were used as the carbon source.

**Figure 6:**
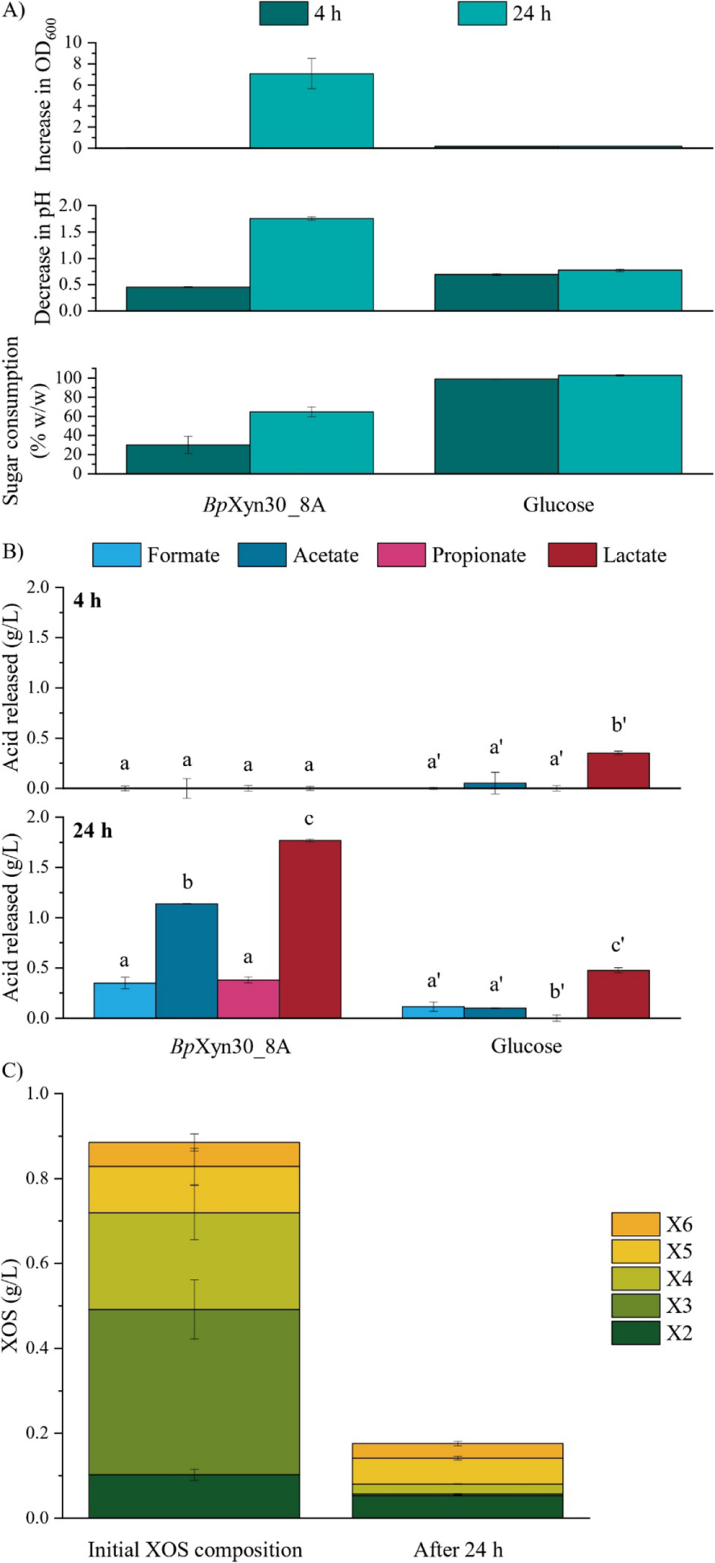
Assessment of the prebiotic activity of XOS produced from glucuronoxylan by *Bp*Xyn30_8A in the growth of *B. adolescentis*. (a) Determination of the parameters of bacterial growth at OD_600_ (upper panel), decrease of the medium pH (middle panel), and cumulative sugar consumption (lower panel; expressed as the percentage of the initial sugar concentration) after incubation of probiotic bacteria with 1 g/L of XOS produced by *Bp*Xyn30_8A or glucose. Experiments were conducted at 37 °C for up to 24 h in static conditions. Wilcoxon and two-sided Mann–Whitney tests were used for significance analysis (*p* < 0.05); no statistical differences were detected for both paired (*p* = 0.25) and unpaired observations (*p* = 0.1). (b) Quantification of short-chain fatty acids by HPLC after 4 h (top panel) and 24 h (lower panel). ANOVA and Tukey tests were used for mean comparison, and different letters refer to statistically different values (*p* < 0.05). (c) Comparison of the linear oligosaccharide concentration (DP: 2–6) in 1 g/L of XOS produced by *Bp*Xyn30_8A added as a carbon source to the bacteria and its composition after 24 h of reaction. Values from the bacterial control were subtracted from the XOS-supplemented samples. Mann–Whitney tests were used for significance analysis (*p* < 0.05); no statistical differences were detected (*p* = 0.1). Data presented are the mean values of time-dependent experiments in triplicate (*n* = 3), and the error bars are ±SD.

Considering that the decrease in pH is associated with the production of SCFAs, we set out to quantify formate, acetate, propionate, lactate, and butyrate produced during fermentation. Acetate and lactate were the main products liberated by *B. adolescentis* (Figure 6b), which is in line with the literature,^62^ however, smaller amounts of formate and propionate were also detected. No butyrate was observed (data not shown). Production of SCFAs increased over time for both sugars, and the decrease in pH proved to be dependent on the concentration of fatty acids. Curiously, solutions supplemented with only 1 g/L glucose revealed lactate as the most significant product, something that was observed when triple the amount of the hexose was employed.^63^ The same work obtained similar results when the carbon source was XOS produced from beechwood glucuronoxylan using GH10 xylanase.

Finally, the composition of X2 to X6 was analyzed in samples containing 1 g/L XOS produced by *Bp*Xyn30_8A (amount added to the reaction) or supernatant after 24 h of fermentation (Figure 6c). Enzymatically produced XOS was composed of almost 90% of linear oligosaccharides with DP from 2 to 6, with xylotriose and xylotetraose being produced in greater quantities. After 24 h of incubation with *B. adolescentis*, X3 and X4 were almost completely depleted. Furthermore, considering that approximately 0.35 g/L of the initial sugar remained unfermented after 24 h, and that 0.18 g/L of this was attributed to linear XOS (DP: 2–6), the remaining ∼0.12 g/L consisted of MeGlcA-substituted (branched) oligosaccharides. This interpretation is supported by the presence of late-eluting peaks in the HPAEC-PAD chromatograms, which are characteristic of branched XOS (Figure S8). These branched structures appeared to be largely unchanged over the incubation period, suggesting they were poorly consumed by *B. adolescentis*. In summary, the bacteria preferentially fermented linear XOS up to 24 h, while branched XOS remained largely unutilized under the tested conditions.

In the context of the human gut, it was determined that the complexity of dietary glycans influences the intestinal microbiota. Rogowski and coauthors (2015)^64^ demonstrated that only simple oligosaccharides liberated by *B. ovatus* grown on birchwood glucuronoxylan and wheat arabinoxylan are consumed by *B. adolescentis*, whereas complex saccharides released from corn glucurono-arabinoxylan cannot be utilized.^64^

*B. adolescentis* primarily uses the ATP-binding cassette (ABC-type) transport systems, which include solute-binding proteins (SBPs) that largely determine the specificity and affinity of the transporter, for the uptake of short xylooligosaccharides (DP: 2–6) derived from xylan degradation products.^65,66^ These SBPs are highly conserved across the genus and have been shown to bind both linear and arabinose-substituted XOS, with affinities within the nanomolar to micromolar range.^67^ Thus, it is not surprising that the probiotic strain used in this study preferentially utilized the small, linear oligosaccharides available in the medium.

Although not every probiotic strain produces butyrate, it is very common in the colon to observe the occurrence of cross-feeding between butyrate-producing and nonproducing strains. A very interesting example is the coculture of *B. adolescentis* and *Eubacterium Hallii*. The former liberates acetate, formate, and lactate as the main byproducts of fermentation, while the latter can only grow on starch and produce butyrate by metabolizing acetate and lactate provided by the *Bifidobacterium* strain.^68^

Overall, the ability of *Bp*Xyn30_8A to generate high amounts of linear XOS distinguishes it from other well-characterized GH30_8 glucuronoxylanases. Despite minor structural variations, the enzyme retains all key residues required for MeGlcA recognition, including a conserved arginine that mediates interactions with ligand residues in the active site. These results underscore the biotechnological potential of *Bp*Xyn30_8A as a versatile enzymatic catalyst for the green production of prebiotic oligosaccharides from xylan-rich plant biomass.

## Supplementary Material



## Notes

Additional biochemical and structural characterization of *Bp*Xyn30_8A, including pH and temperature profiles, residual activity, enzymatic cleavage pattern, different views of the crystallographic structure, and putative interactions with the substrates (PDF)
