# Supplementary material for: Enzymatic Production of Prebiotic Xylooligosaccharides Using a Bacillus pumilus GH30_8 Glucuronoxylanase: Structural Basis of Glucuronoxylan Recognition and Hydrolysis
Source: J Agric Food Chem. 2026 Feb 3;74(6):5417–30. doi: 10.1021/acs.jafc.5c07569 (PMC12921914; doi:10.1021/acs.jafc.5c07569)
Supplement: Supplementary file 1 [file jf5c07569_si_001.pdf]

Supporting information

Enzymatic production of prebiotic xylooligosaccharides using a *Bacillus pumilus* GH30_8 glucuronoxylanase: Structural basis of glucuronoxylan recognition and hydrolysis

Milena Moreira Vacilotto[1], Vanessa de Oliveira Arnoldi Pellegrini[1], Evandro Ares de Araujo[2], Marcelo V. Liberato[1] and Igor Polikarpov[1*]

[1]Instituto de Física de São Carlos, Universidade de São Paulo, Avenida Trabalhador São-carlense 400, 13566-590 São Carlos, SP, Brazil.

[2]Centro Nacional de Pesquisa em Energia e Materiais, Giuseppe Máximo Scolfaro 10000, 13083-100 Campinas, SP, Brazil.

*Correspondent author: ipolikarpov@ifsc.usp.br

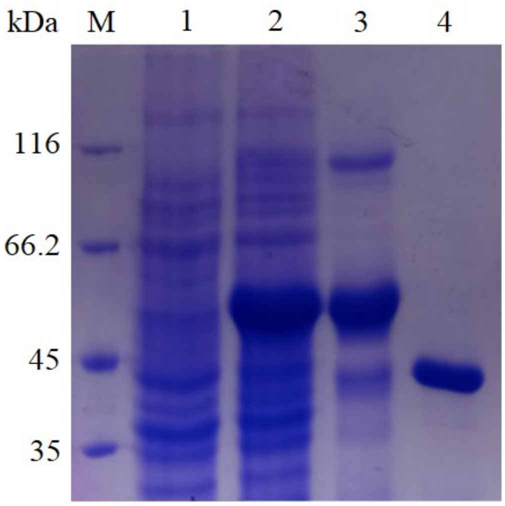

Figure S1. SDS-PAGE gel of *Bp*Xyn30A production and purification steps. M: low molecular weight marker; 1: cell culture before induction with IPGT; 2: cell culture after 16 h of protein production; 3: *Bp*Xyn30A (58.7 kDa) after the first purification using nickel affinity chromatography; 4: non-fused xylanase (44.4 kDa) after second purification using Ni^{2+} affinity chromatography.

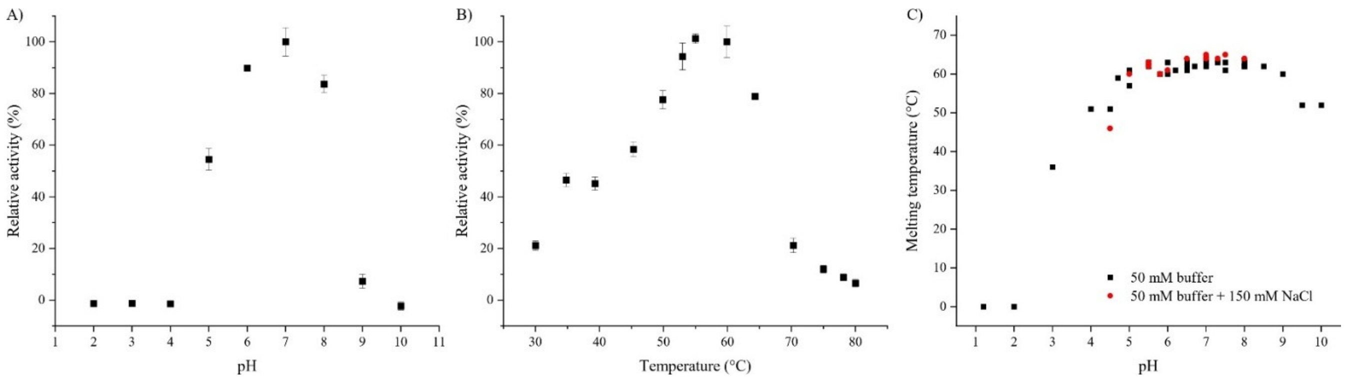

Figure S2. Optimum conditions for *Bp*Xyn30A enzymatic assays and stability. Optimal pH (a) and temperature (b) of the xylanase were obtained by using 50 mM ABF buffers with pHs from 2 to 10 and 50 °C for the former and by fixing the pH in 7 and varying the temperature from 20 to 80 °C for the latter. (c) DSF assay was used to evaluate the best condition for the enzyme handing, and 48 different 50 mM buffers with pHs ranging from 1.2 to 10 containing or not 150 mM NaCl were tested. Data presented are the mean value of three independent experiments (*n* = 3), and error bars are ± SD.

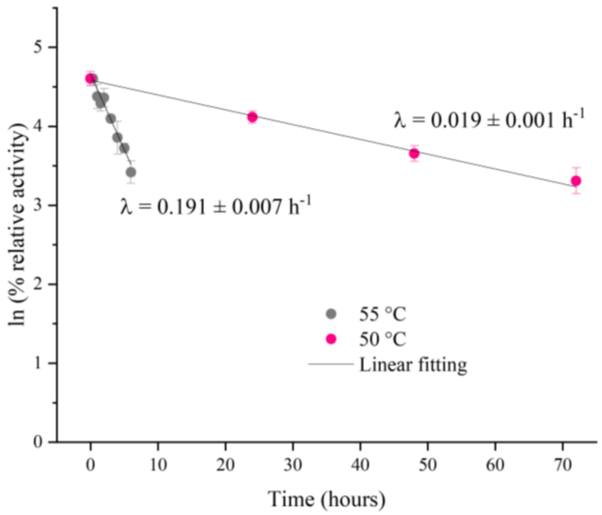

Figure S3. Natural logarithm of *Bp*Xyn30A residual activity (in percentage relative to maximum activity) as a function of time. The enzyme was incubated in 50 mM Tris-HCl pH 7 at 50 °C or 55 °C, and aliquots were removed over time to test its activity using the DNS assay. Data presented are the mean value of three independent experiments ($n = 3$), and error bars are ± SD.

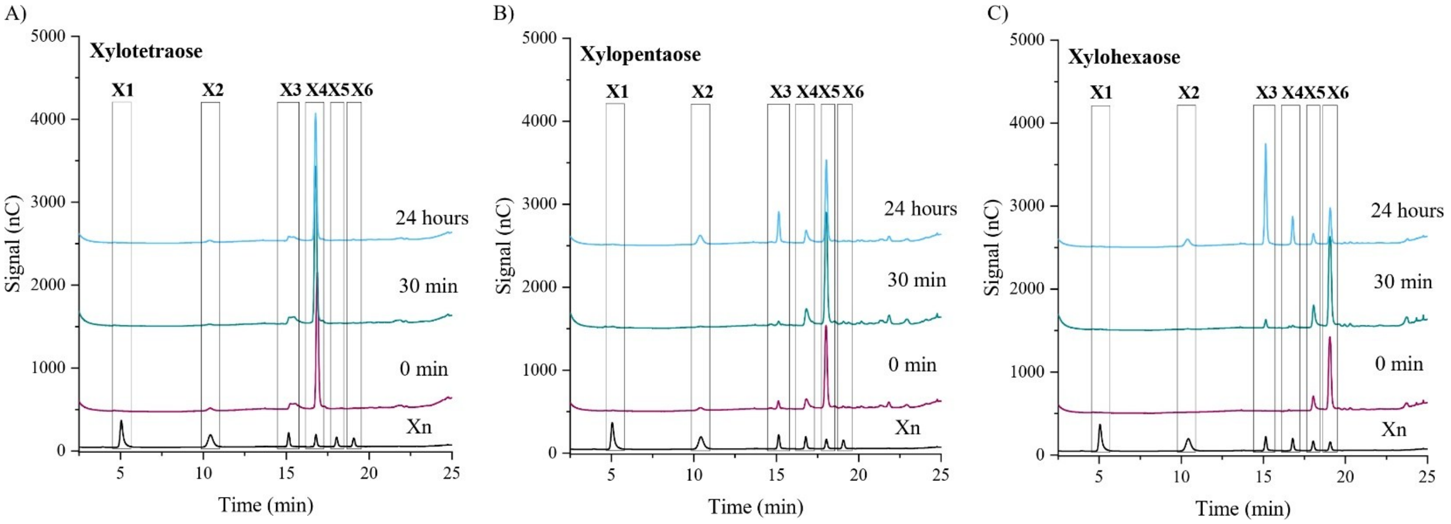

Figure S4. Enzymatic cleavage pattern of *Bp*Xyn30_8A in the presence of 0.5 mg/mL of (a) xylotetraose, (b) xylopentaose or (c) xylohexaose. Reactions were carried out with180 nM *Bp*Xyn30_8A in 20 mM Tris-HCl buffer pH 7 and maintained at 50 °C for up to 24 h. Samples were analyzed by HPAEC-PAD. Marked standards represent, Xn: xylose and xylooligosaccharides (DP: 2 – 6), X3: xylotetraose, X4: xylotetraose, X5: xylopentaose, X6: xylohexaose.

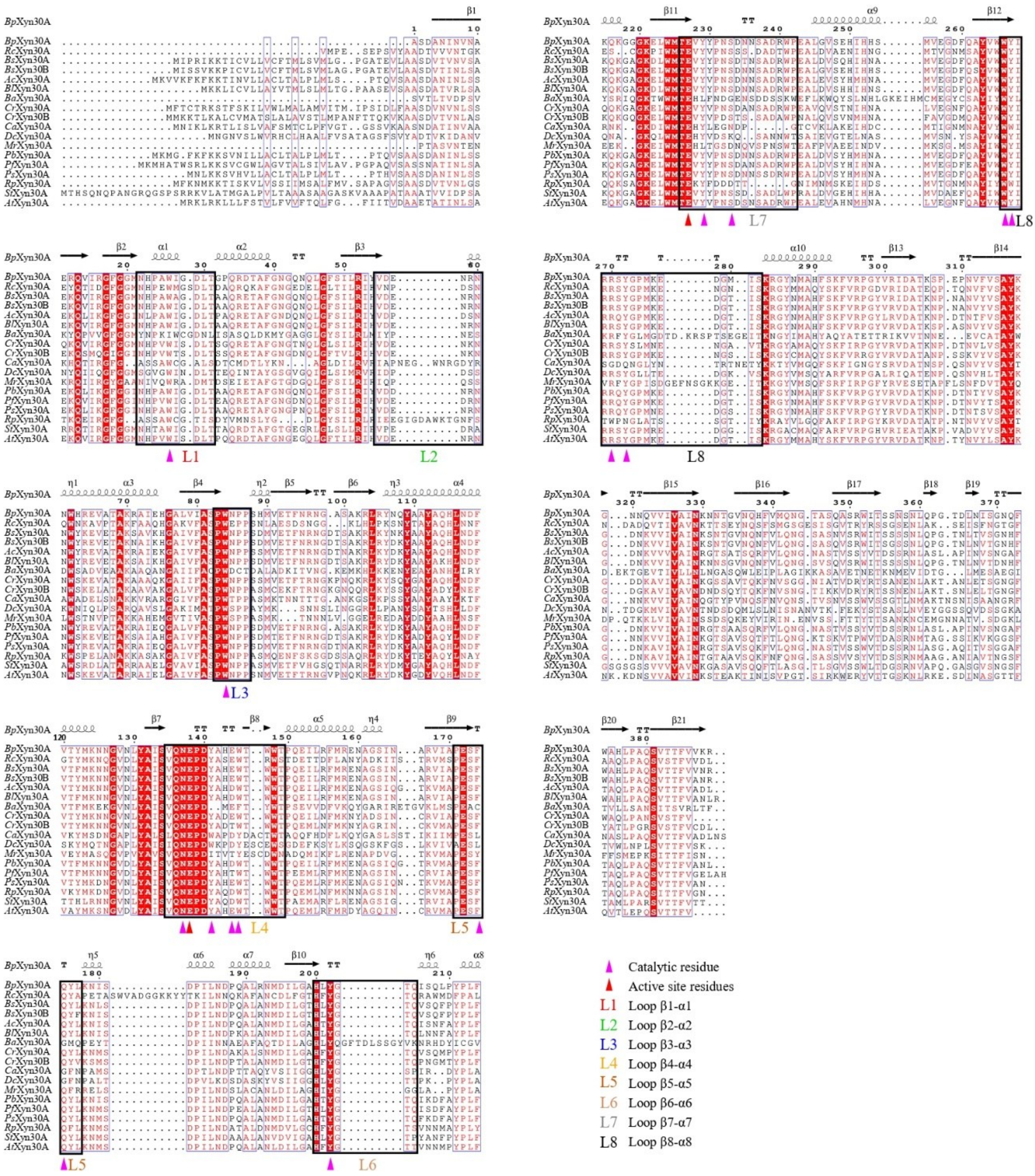

Figure S5. Alignment of *Bp*Xyn30A and seventeen other GH30_8 xylanases. Catalytic residues and active site residues are indicated by red and pink triangles, respectively. Loops surrounding the catalytic site were numbered and colored according to [1] as follows: L1 in red (loop β1-α1), L2 in green (loop β2-α2), L3 in blue (loop β3-α3), L4 in yellow (loop β4-α4), L5 in orange (loop β5-α5), L6 in salmon (loop β6-α6), L7 in grey (loop β7-α7) and L8 in black (loop β8-α8).

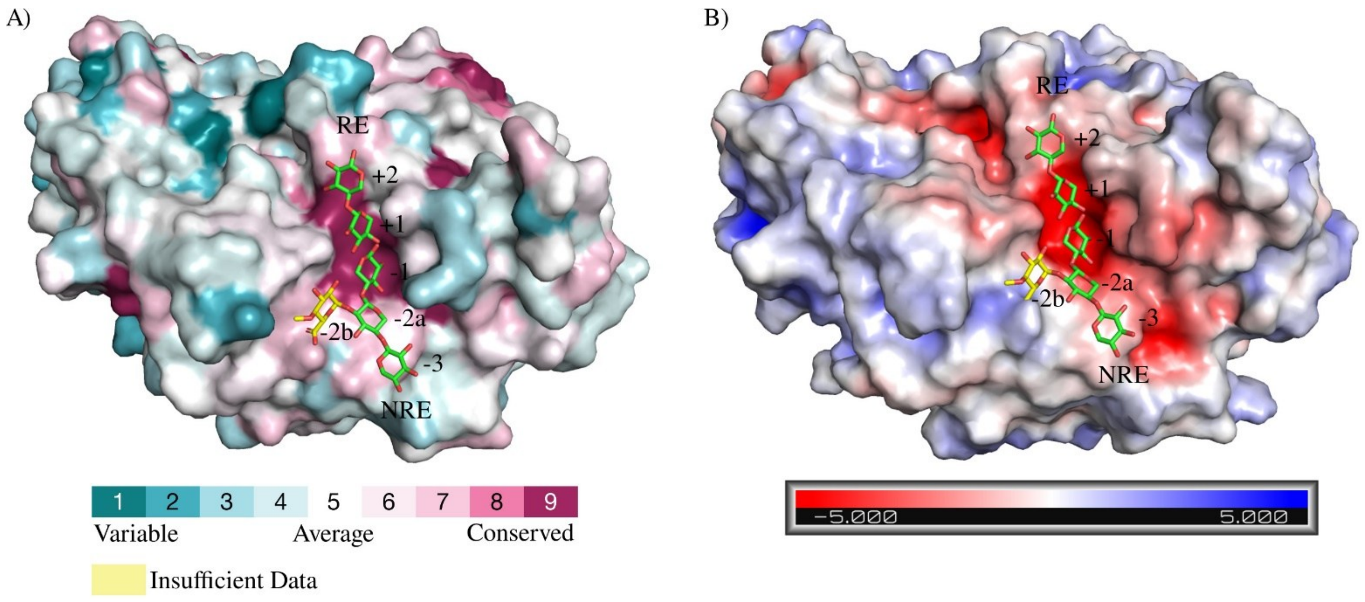

Figure S6. Front view of the (a) conservation map and (b) Poisson-Boltzmann electrostatic potential of *Bp*Xyn30A. $MeGlcA^4X5$ ligand was modeled based on the structures of *Dc*Xyn30A in complex with $MeGlcA^2X3$ (PDB entry 2Y24) and *At*Xyn30A in complex with xylobiose (PDB entry 5A6L). RE and NRE stands for reducing end and non-reducing end, respectively.

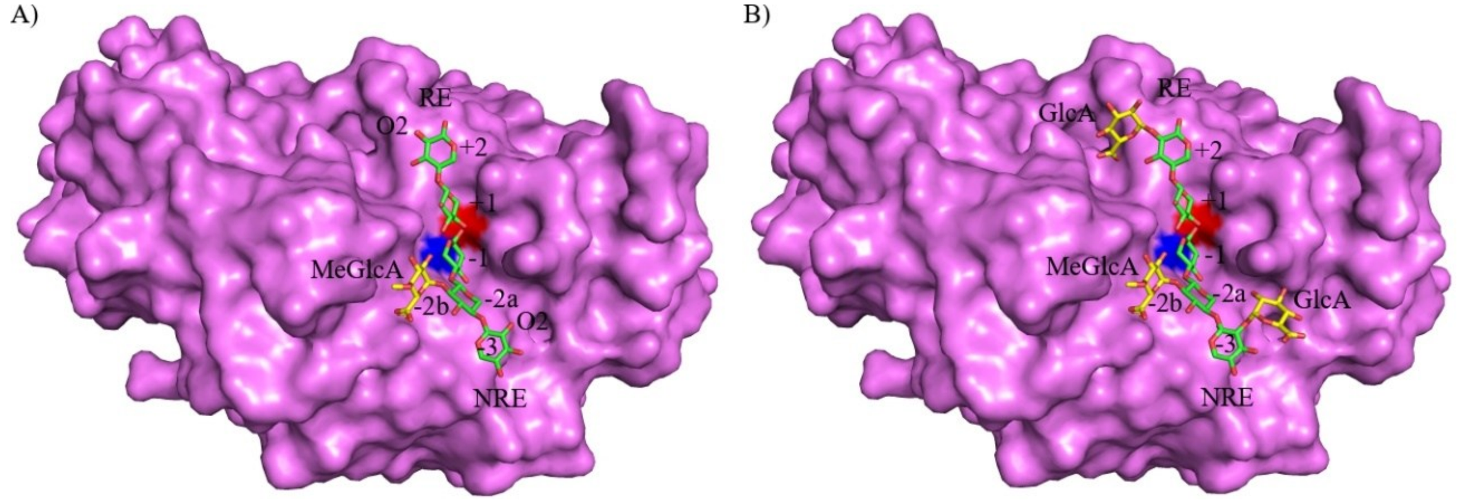

Figure S7. *Bp*Xyn30A crystallographic structure in the presence of (a) MeGlcA^{4}X5 and (b) GlcA^{1,5}MeGlcA^{4}X5. Ligands were obtained from the structures of *Dc*Xyn30A in complex with MeGlcA^{2}X3 (PDB entry 2Y24) and *At*Xyn30A in complex with xylobiose (PDB entry 5A6L). GlcA decorations were manually added to the chimeric substrate using the GLYCAM-Web server (https://glycam.org/cb/). Catalytic acid/base residue (Glu138) is colored in red and nucleophile (Glu227) in blue. RE and NRE stands for reducing end and non-reducing end, respectively.

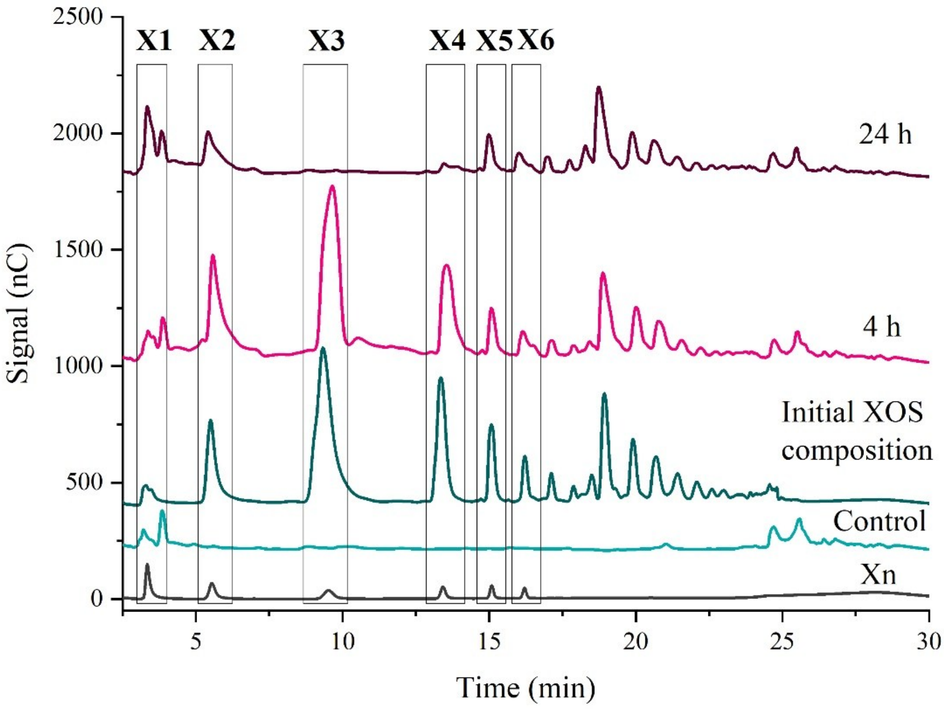

Figure S8. HPAEC-PAD chromatograms of XOS consumption by *B. adolescentis*. The control reaction contained the probiotic bacteria but no XOS, whereas initial XOS composition is made up of 1 g/L of beechwood xylan hydrolysate generated by *Bp*Xyn30A. Experiments were conducted at 37 °C under static conditions for up to 24 h. Marked standards represent, Xn: xylose and xylooligosaccharides (DP: 2 – 6), X3: xylotetraose, X4: xylotetraose, X5: xylopentaose, X6: xylohexaose.

Supplementary Tables

Table S1. Data Collection and Refinement Statistics for *Bp*Xyn30_8A. Statistics for the highest-resolution shell are shown in parentheses.

Parameter	*Bp*Xyn30_8A
Wavelength (Å)	0.9772
Resolution range (Å)	46.91 – 2.16 (2.237 – 2.16)
Space group	$P\ 2_1\ 2_1\ 2_1$
Unit cell parameters (Å, °)	59.084, 101.484, 158.689; 90, 90, 90
Total reflections	102,540 (9,745)
Unique reflections	51,299 (4,885)
Multiplicity	2.0 (2.0)
Completeness (%)	98.54 (95.63)
Mean I/σ(I)	8.58 (1.27)
Wilson B-factor (Å^2)	35.05
R-merge	0.06205 (0.5742)
R-meas	0.08775 (0.8121)
R-pim	0.06205 (0.5742)
$CC_{1/2}$	0.996 (0.489)
CC*	0.999 (0.81)
R-work	0.1786 (0.2912)
R-free	0.2106 (0.3176)
CC(work)	0.971 (0.688)
CC(free)	0.962 (0.676)
Protein residues per chain	778
RMS(bonds) (Å)	0.002
RMS(angles) (°)	0.53
Ramachandran favored (%)	97.03
Ramachandran allowed (%)	2.71
Ramachandran outliers (%)	0.26
Rotamer outliers (%)	0.76
Clashscore	3.35
Average B-factor (Å^2)	38.37
Macromolecules	38.15
Solvent	42.06
PDB ID	9O5H

Table S2. GH30_8 xylanases used for the sequence alignment. Names of the enzymes were standardized using their organism of origin as prefix and a suffix (A or B) according to the year of publication.

Organism / Name	Genbank ID	Reference
*Acetivibrio thermocellus ATCC 27405 / At*Xyn30A	ABN54208.1	[2,3]
Aeromonas caviae ME-1 / *Ac*Xyn30A	AAB63573.1	[4]
Bacillus licheniformis SVD1 / *Bl*Xyn30A	BAL45490.1	[5]
Bacillus sp. BP7 / *Bs*Xyn30B	ADM15019.1	[6]
Bacillus subtilis str. 168 / *Bs*Xyn30A	CAA97612.1	[7,8]
*Bacteroides ovatus A*TCC 8483 / *Ba*Xyn30A	ALJ48333.1	[9]
Cellulosilyticum ruminicola CGMCC 1.5065 / *Cr*Xyn30A	ACZ98597.1	[10]
Cellulosilyticum ruminicola MMBC-1 / *Cr*Xyn30B	UZE89494.1	[11]
Clostridium acetobutylicum ATCC 824 / *Ca*Xyn30A	AAK76864.1	[12]
Dickeya chrysanthemi D1 / *Dc*Xyn30A	AAB53151.1	[13,14]
*Melioribacter roseus P3M-2 / Mr*Xyn30A	AFN75320.1	[15]
*Paenibacillus barcinonensis BP-23 / Pb*Xyn30A	AEY82463.1	[16]

*Paenibacillus favisporus CC02-N2 / Pf*Xyn30A	AHA38215.1	[17]
*Paenibacillus sp. W-61 / Ps*Xyn30A	BAA13641.1	[18]
*Ruminiclostridium papyrosolvens DSM 2782 / Rp*Xyn30A	EGD48159.1	[19]
Ruminococcus champanellensis JCM 17042 / *Rc*Xyn30A	WP_147645661.1	[1]
*Streptomyces turgidiscabies C56 / St*Xyn30A	WP_006381841.1	[20]

References

(1) Vacilotto, M. M.; de Araujo Montalvão, L.; Pellegrini, V. de O. A.; Liberato, M. V.; de Araujo, E. A.; Polikarpov, I. Two-Domain GH30 Xylanase from Human Gut Microbiota as a Tool for Enzymatic Production of Xylooligosaccharides: Crystallographic Structure and a Synergy with GH11 Xylosidase. *Carbohydr. Polym.* **2024**, *337*, 122141. https://doi.org/10.1016/j.carbpol.2024.122141.

(2) Verma, A. K.; Goyal, A. A Novel Member of Family 30 Glycoside Hydrolase Subfamily 8 Glucuronoxylan Endo-β-1,4-Xylanase (CtXynGH30) from Clostridium Thermocellum Orchestrates Catalysis on Arabinose Decorated Xylans. *J. Mol. Catal. B Enzym.* **2016**, *129*, 6–14. https://doi.org/10.1016/j.molcatb.2016.04.001.

(3) Freire, F.; Verma, A.; Bule, P.; Alves, V. D.; Fontes, C. M. G. A.; Goyal, A.; Najmudin, S. Conservation in the Mechanism of Glucuronoxylan Hydrolysis Revealed by the Structure of Glucuronoxylan Xylanohydrolase (Ct Xyn30A) from Clostridium Thermocellum. *Acta Crystallogr. Sect. D Struct. Biol.* **2016**, *72*

(11), 1162–1173. https://doi.org/10.1107/S2059798316014376.

(4) Suzuki, T.; Ibata, K.; Hatsu, M.; Takamizawa, K.; Kawai, K. Cloning and Expression of a 58-KDa Xylanase VI Gene (XynD) of Aeromonas Caviae ME-1 in Escherichia Coli Which Is Not Categorized as a Family F or Family G Xylanase. *J. Ferment. Bioeng.* **1997**, *84* (1), 86–89. https://doi.org/10.1016/S0922-338X(97)82792-4.

(5) Sakka, M.; Tachino, S.; Katsuzaki, H.; van Dyk, J. S.; Pletschke, B. I.; Kimura, T.; Sakka, K. Characterization of Xyn30A and Axh43A of Bacillus Licheniformis SVD1 Identified by Its Genomic Analysis. *Enzyme Microb. Technol.* **2012**, *51* (4), 193–199. https://doi.org/10.1016/j.enzmictec.2012.06.003.

(6) Gallardo, O.; Fernández-Fernández, M.; Valls, C.; Valenzuela, S. V.; Roncero, M. B.; Vidal, T.; Díaz, P.; Pastor, F. I. J. Characterization of a Family GH5 Xylanase with Activity on Neutral Oligosaccharides and Evaluation as a Pulp Bleaching Aid. *Appl. Environ. Microbiol.* **2010**, *76* (18), 6290–6294. https://doi.org/10.1128/AEM.00871-10.

(7) St. John, F. J.; Rice, J. D.; Preston, J. F. Characterization of XynC from Bacillus Subtilis Subsp. Subtilis Strain 168 and Analysis of Its Role in Depolymerization of Glucuronoxylan. *J. Bacteriol.* **2006**, *188* (24), 8617–8626. https://doi.org/10.1128/JB.01283-06.

(8) St John, F. J.; Hurlbert, J. C.; Rice, J. D.; Preston, J. F.; Pozharski, E. Ligand Bound Structures of a Glycosyl Hydrolase Family 30 Glucuronoxylan Xylanohydrolase. *J. Mol. Biol.* **2011**, *407* (1), 92–109. https://doi.org/10.1016/j.jmb.2011.01.010.

(9) Rogowski, A.; Briggs, J. A.; Mortimer, J. C.; Tryfona, T.; Terrapon, N.; Lowe, E. C.; Baslé, A.; Morland, C.; Day, A. M.; Zheng, H.; Rogers, T. E.; Thompson, P.;

Hawkins, A. R.; Yadav, M. P.; Henrissat, B.; Martens, E. C.; Dupree, P.; Gilbert, H. J.; Bolam, D. N. Glycan Complexity Dictates Microbial Resource Allocation in the Large Intestine. *Nat. Commun.* **2015**, *6* (1), 7481. https://doi.org/10.1038/ncomms8481.

(10) Cai, S.; Li, J.; Hu, F. Z.; Zhang, K.; Luo, Y.; Janto, B.; Boissy, R.; Ehrlich, G.; Dong, X. Cellulosilyticum Ruminicola , a Newly Described Rumen Bacterium That Possesses Redundant Fibrolytic-Protein-Encoding Genes and Degrades Lignocellulose with Multiple Carbohydrate- Borne Fibrolytic Enzymes. *Appl. Environ. Microbiol.* **2010**, *76* (12), 3818–3824. https://doi.org/10.1128/AEM.03124-09.

(11) Liu, J.; Zhu, J.; Xu, Q.; Shi, R.; Liu, C.; Sun, D.; Liu, W. Functional Identification of Two Novel Carbohydrate-Binding Modules of Glucuronoxylanase CrXyl30 and Their Contribution to the Lignocellulose Saccharification. *Biotechnol. Biofuels Bioprod.* **2023**, *16* (1), 40. https://doi.org/10.1186/s13068-023-02290-7.

(12) St John, F. J.; Dietrich, D.; Crooks, C.; Balogun, P.; de Serrano, V.; Pozharski, E.; Smith, J. K.; Bales, E.; Hurlbert, J. A Plasmid Borne, Functionally Novel Glycoside Hydrolase Family 30 Subfamily 8 Endoxylanase from Solventogenic Clostridium. *Biochem. J.* **2018**, *475* (9), 1533–1551. https://doi.org/10.1042/BCJ20180050.

(13) Larson, S. B.; Day, J.; Barba de la Rosa, A. P.; Keen, N. T.; McPherson, A. First Crystallographic Structure of a Xylanase from Glycoside Hydrolase Family 5: Implications for Catalysis ,. *Biochemistry* **2003**, *42* (28), 8411–8422. https://doi.org/10.1021/bi034144c.

(14) Šuchová, K.; Kozmon, S.; Puchart, V.; Malovíková, A.; Hoff, T.; Mørkeberg

Krogh, K. B. R.; Biely, P. Glucuronoxylan Recognition by GH 30 Xylanases: A Study with Enzyme and Substrate Variants. *Arch. Biochem. Biophys.* **2018**, *643*, 42–49. https://doi.org/10.1016/j.abb.2018.02.014.

(15) Rakitin, A. L.; Ermakova, A. Y.; Ravin, N. V. Novel Endoxylanases of the Moderately Thermophilic Polysaccharide-Degrading Bacterium Melioribacter Roseus. *J. Microbiol. Biotechnol.* **2015**, *25* (9), 1476–1484. https://doi.org/10.4014/jmb.1501.01061.

(16) Valenzuela, S. V.; Diaz, P.; Pastor, F. I. J. Modular Glucuronoxylan-Specific Xylanase with a Family CBM35 Carbohydrate-Binding Module. *Appl. Environ. Microbiol.* **2012**, *78* (11), 3923–3931. https://doi.org/10.1128/AEM.07932-11.

(17) Padilha, I. Q. M.; Valenzuela, S. V; Grisi, T. C. S. L.; Díaz, P.; de Araújo, D. A. M.; Pastor, F. I. J. A Glucuronoxylan-Specific Xylanase from a New Paenibacillus Favisporus Strain Isolated from Tropical Soil of Brazil. *Int. Microbiol.* **2014**, *17* (3), 175–184. https://doi.org/10.2436/20.1501.01.220.

(18) Dung, N. V.; Vetayasuporn, S.; Kamio, Y.; Abe, N.; Kaneko, J.; Izaki, K. Purification and Properties of β-1,4-Xylanases 2 and 3 from Aeromonas Caviae W-61. *Biosci. Biotechnol. Biochem.* **1993**, *57* (10), 1708–1712. https://doi.org/10.1271/bbb.57.1708.

(19) St John, F. J.; Dietrich, D.; Crooks, C.; Pozharski, E.; González, J. M.; Bales, E.; Smith, K.; Hurlbert, J. C. A Novel Member of Glycoside Hydrolase Family 30 Subfamily 8 with Altered Substrate Specificity. *Acta Crystallogr. Sect. D Biol. Crystallogr.* **2014**, *70* (11), 2950–2958. https://doi.org/10.1107/S1399004714019531.

(20) Maehara, T.; Yagi, H.; Sato, T.; Ohnishi-Kameyama, M.; Fujimoto, Z.; Kamino, K.; Kitamura, Y.; St. John, F.; Yaoi, K.; Kaneko, S. GH30 Glucuronoxylan-

Specific Xylanase from Streptomyces Turgidiscabies C56. *Appl. Environ. Microbiol.* **2018**, *84* (4). https://doi.org/10.1128/AEM.01850-17.
